# Regional Variation in the Prevalence of *E. coli* O157 in Cattle: A Meta-Analysis and Meta-Regression

**DOI:** 10.1371/journal.pone.0093299

**Published:** 2014-04-01

**Authors:** Md. Zohorul Islam, Alfred Musekiwa, Kamrul Islam, Shahana Ahmed, Sharmin Chowdhury, Abdul Ahad, Paritosh Kumar Biswas

**Affiliations:** 1 Department of Microbiology, Chittagong Veterinary and Animal Sciences University, Chittagong, Bangladesh; 2 School of Mathematics, Statistics and Computer Science, University of KwaZulu-Natal, Pietermaritzburg, South Africa; 3 Chittagong Veterinary Laboratory, Chittagong, Bangladesh; 4 Department of Pathology and Parasitology, Chittagong Veterinary and Animal Sciences University, Chittagong, Bangladesh; Iowa State University, United States of America

## Abstract

**Background:**

*Escherichia coli* O157 (EcO157) infection has been recognized as an important global public health concern. But information on the prevalence of EcO157 in cattle at the global and at the wider geographical levels is limited, if not absent. This is the first meta-analysis to investigate the point prevalence of EcO157 in cattle at the global level and to explore the factors contributing to variation in prevalence estimates.

**Methods:**

Seven electronic databases- CAB Abstracts, PubMed, Biosis Citation Index, Medline, Web of Knowledge, Scirus and Scopus were searched for relevant publications from 1980 to 2012. A random effect meta-analysis model was used to produce the pooled estimates. The potential sources of between study heterogeneity were identified using meta-regression.

**Principal findings:**

A total of 140 studies consisting 220,427 cattle were included in the meta-analysis. The prevalence estimate of EcO157 in cattle at the global level was 5.68% (95% CI, 5.16–6.20). The random effects pooled prevalence estimates in Africa, Northern America, Oceania, Europe, Asia and Latin America-Caribbean were 31.20% (95% CI, 12.35–50.04), 7.35% (95% CI, 6.44–8.26), 6.85% (95% CI, 2.41–11.29), 5.15% (95% CI, 4.21–6.09), 4.69% (95% CI, 3.05–6.33) and 1.65% (95% CI, 0.77–2.53), respectively. Between studies heterogeneity was evidenced in most regions. World region (p<0.001), type of cattle (p<0.001) and to some extent, specimens (p = 0.074) as well as method of pre-enrichment (p = 0.110), were identified as factors for variation in the prevalence estimates of EcO157 in cattle.

**Conclusion:**

The prevalence of the organism seems to be higher in the African and Northern American regions. The important factors that might have influence in the estimates of EcO157 are type of cattle and kind of screening specimen. Their roles need to be determined and they should be properly handled in any survey to estimate the true prevalence of EcO157.

## Introduction

Enterohemorrhagic *Escherichia coli* O157 (EHEC O157), also known as verocytotoxin producing or shiga toxin producing EcO157, is a very important and world-wide reported food-borne pathogen. It causes hemorrhagic colitis with some severe sequelae including hemolytic uremic syndrome which is caused by the effect of shiga toxin produced by the organism that acts on kidney, intestine and other parenchymatous organs [1]. Cattle are the natural reservoir of it [1] contributing as a major source for human infections [2]–[6]. The public health concern of EcO157 came to light at first after its first outbreak reported in the USA in 1982 [7]. Although the organism does not produce any clinical illness in their natural reservoir, it can produce a broad spectrum of clinical abnormalities in humans including mild diarrhea, hemorrhagic colitis (HC), hemolytic uremic syndrome (HUS), bloody diarrhea and thrombotic thrombocytopenic purpura (TTP) [8], [9]. Harboring of EcO157 in cattle is a significant concern for public health because of their transmitting capability to humans through contaminated foods and water with feces from cattle [10]–[13]. A wide range of prevalence estimates ranging from 0.1% to 62% of EcO157 in cattle was reported worldwide [12], [14]–[19].

The inconsistent prevalence estimates of EcO157 reported in cattle in various geographical locations might be, to some extent, due to variable methodological modus operandi to identify the organism, such as sampling strategy, type of samples, enrichment procedures, immunomagnetic separation and cultural media of choice. Therefore, the factors that contribute to the variability in the detection of the organism and thus in the prevalence estimate need to be identified by analyzing the available published reports.

The aims of the study were to illustrate the prevalence estimates of EcO157 in cattle both at the global and at different geographical levels, and to generate empirical evidence on the sources/factors contributing to between study heterogeneity.

## Methods

### Search strategy

A systematic search strategy was used to identify all published studies reporting prevalence of EcO157 in cattle. Seven electronic databases-CAB Abstracts, PubMed, Biosis Citation Index, Medline, Web of Knowledge, Scirus and Scopus were searched for relevant studies published from 1980 to 2012. Two authors (MZI and KI) were assigned to search the databases. The search terms were adapted from Seargent et al. [20] and grouped into three categories: outcome, population and descriptive. Modified search terms are presented in Table 1.

**Table 1:** Algorithm for electronic database search to find published reports on prevalence of *E. coli* O157 in cattle.

Search term	Boolean keywords
**Descriptive term**	Prevalence OR Incidence OR frequency OR occurrence OR Detection OR Identification OR Isolation OR characterization OR Investigation
**Population term**	Escherichia coli O157 OR E coli O157 OR O157 OR shiga toxin producing Escherichia coli O157 OR STEC O157 OR VTEC O157 OR verocytotoxin producing Escherichia coli O157 OR EHEC O157 OR Enterohaemorrhagic Escherichia coli O157 OR Enterohemorrhagic Escherichia coli O157 OR VTEC OR STEC OR EHEC OR Enterohemorragic OR Enterohaemorrhagic OR Enterohemorrhagic OR Enterohaemorragic
**Outcome term**	Cattle OR Bovine OR ruminant OR dairy OR Beef OR cow OR veal OR Calf OR calves OR heifer OR steer OR feedlot OR Bull OR Bullock OR yearling

The Boolean operator “AND” was used to combine the categories and “OR” was used to join the terms within each category respectively. Search terms and keywords were altered as per specification of individual databases. No language restrictions were applied. The reference lists of retrieved articles were searched manually to identify all potential studies so that no articles have been missed by the electronic searches. Database search was undertaken on the 12^th^ and 13^th^ of February, 2013.

### Inclusion and exclusion criteria

Two authors (MZI and KI) independently screened the titles and abstracts of search results to identify potential studies. Initial screening was performed according to some predefined inclusion and exclusion criteria based on research hypothesis. Full text articles were retrieved if they met the inclusion criteria.

Any article to be included in the meta-analysis had the following inclusion criteria: it had to be published between 1980 and 2012, reported with animal level prevalence data, any kind of cattle population from any place in the globe, type of specimens as intestinal content, feces, rectal swab and/or other enteric substances from cattle and the identity of bacterial isolates as EcO157 (H7 or not) by any of the recognized techniques: latex agglutination test, slide agglutination test, serotyping, and/or PCR for *rfbO157* gene. Cross-sectional, case-control, longitudinal and cohort studies were eligible for inclusion. Studies were excluded if they had duplicate population group, insufficient prevalence data, farm level prevalence, pooled samples, and failing to meet the inclusion criteria mentioned above. Unpublished studies, conference abstracts, experimental and intervention studies were not included in this meta-analysis.

### Data extraction

A pretested data extraction spreadsheet was developed and evaluated (File S1). Full text articles were screened independently by two authors (MZI and KI) and data were also extracted independently by them. Any disagreements between the two authors were resolved by consensus and/or cross-checking with a third author (SA). Data were extracted on first author, study location, year of publication, prevalence of EcO157, study date, study population, type of specimens, origin of sampled cattle, type of cattle, health status of animal, methods of pre-enrichment, isolation media and methods of confirmation. The characteristics of the studies included are mentioned in File S2.

### Data analysis

All the data were analyzed using STATA 12.0 (StataCorp LP, College Station, Texas, USA). Between study variations in the prevalence of EcO157 in cattle were estimated using Chi-square test to evaluate whether the variation between studies exceeds the expected by chance and calculating *I^2^* statistic which represents the proportion of total variation in effect estimates across the studies attributable to heterogeneity rather than by chance [21]. Only crude estimates of prevalence were used in this study. Prevalence was estimated by the number of cases divided by the total number of cattle in the sample, and expressed as a percent. The 95% confidence intervals (CI) were calculated using the standard formula for a proportion: p±1.96*sqrt[p*(100-p)/n]. In cases where the lower limit of the 95% CI was negative, we set the value to zero to avoid negative prevalence. The point estimates from separate studies were pooled using a random effect meta-analysis model. The meta-analysis was performed using STATA command ‘metan’ specifying random. The study estimates were combined by applying the DerSimonian-Laird random effects method [22]. The random effect model was selected because of high degree of heterogeneity (*I^2^*>75%) between the studies. Potential sources of between study heterogeneity were recognized from a group of possibly related variables by using a meta-regression model. Eight potential sources of heterogeneity were examined: region (world region), specimens (rectal swab/feces from rectum/feces from intestine/mixed type/voided feces), origin of sampled cattle (animal pen/others/slaughter house), type of cattle (dairy/beef cattle/others/feedlot), health status (healthy/not mentioned/diseased/mixed type), pre-enrichment (with inhibitors/others/without inhibitors/no pre-enrichment), immunomagnetic separation (yes/no/others) and isolation media (CT-SMAC, CHROMagar O157, MacConkey, SMAC, others). Initially a univariable meta-regression model was built to examine the association between a selected variable and prevalence of EcO157 in cattle separately. A random effect multivariable meta-regression model was built to assess the integrated association of prevalence with the variables. All the variables were tested separately in univariable analysis to identify their contribution to between study heterogeneity. Variables with p<0.2 in the univariable analysis were included into the multivariable model. The variable ‘region’ was at first entered into the multivariable meta-regression model. Manual forward selection, starting with variables that were more strongly associated with prevalence in univariable analyses, was applied and variables were retained in the multivariable model if p<0.2.The variables that were highly significant (p<0.05) in multivariable meta-regression model were considered as building blocks for interaction terms. The extent of publication bias was assessed using a funnel plot and the sources of funnel plot asymmetry were also tested to identify the small study effects.

### Maintenance of study standard

The study was conducted following the guidelines for reporting meta-analysis of observational studies (MOOSE Statement) [23]. In addition, Preferred Reporting Items for Systematic Reviews and Meta-Analyses (PRISMA) statements [24] and PRISMA 2009 checklist (Checklist S1) were followed to maintain the study standard. There is no published protocol for this meta-analysis. We did not assess the quality of individual study, because this meta-analysis was based on observational findings of prevalence studies and was not proper for quantitative synthesis, but we assessed publication bias that may affect the cumulative evidence.

## Results

**Figure 1:**
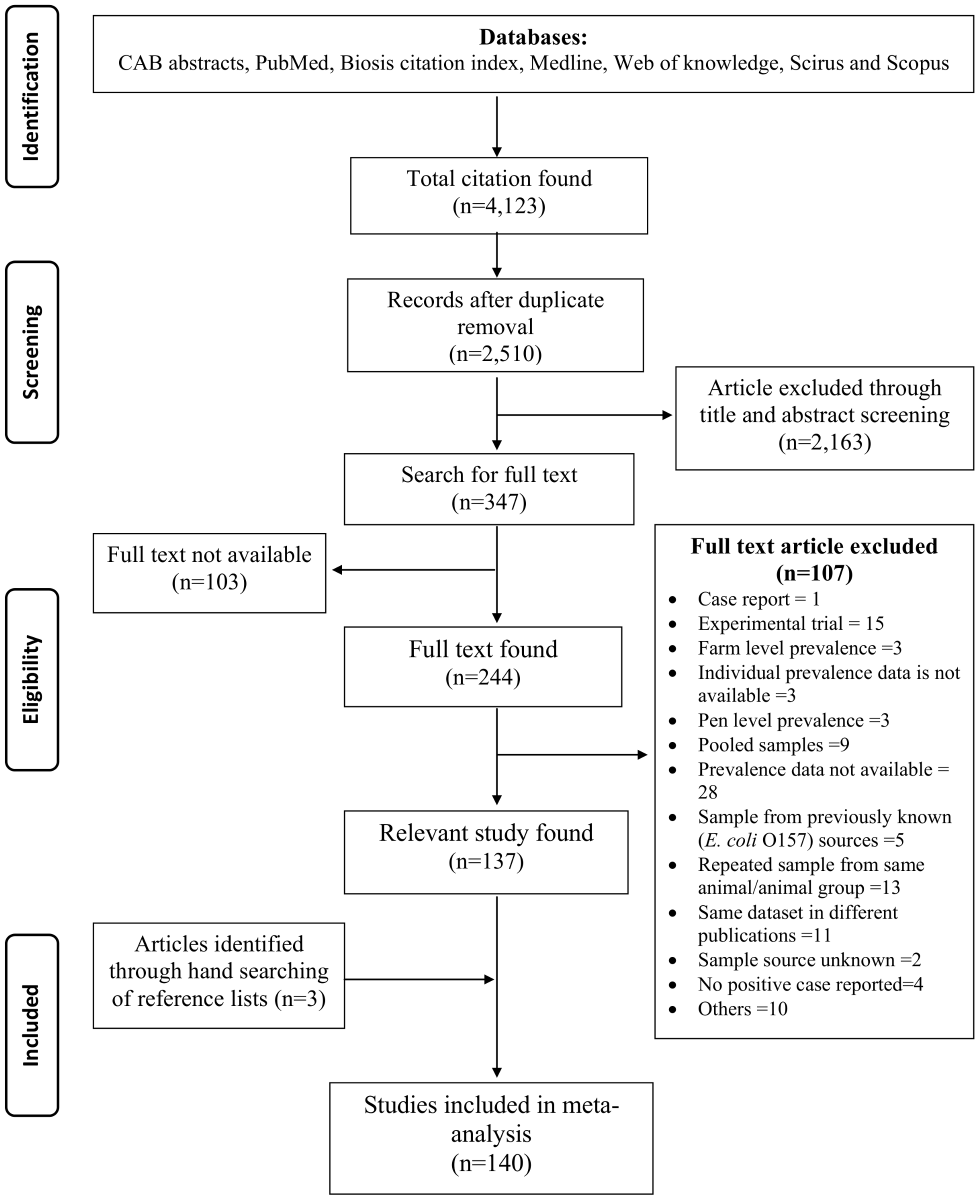
Flow diagram of study selection for inclusion in the meta-analysis.

In the initial search, 2,510 potentially relevant studies were identified. After primary screening of titles and abstracts, 347 articles were selected for full text search. Among them 244 full text articles were retrieved to check the eligibility, of which 137 were included (Figure 1). Additional three eligible studies were identified by hand searches of reference lists of the selected articles. Therefore, finally, a total of 140 articles were included in the meta-analysis (File S3). Lists of excluded full text articles along with the reasons for their exclusion are provided in “File S4”. Description of characteristics of each study reporting the prevalence of EcO157 in cattle is shown in “File S2”.

All the studies included represent data from 38 countries across the globe. The highest number (53) of studies (n = 88,643) was reported from Europe covering 16 countries. In Europe, 14 studies were from the United Kingdom [18], [25]–[37], seven from each of Ireland [38]–[44] and Italy [45]–[51], four from each of France [52]–[55] and Turkey [56]–[59], two from each of Norway [60], [61], Serbia [62], [63], Spain [3], [64], Sweden [65], [66], Switzerland [67], [68] and the Netherlands [69], [70], and one from each of Belgium [71], Czeck Republic [72], Denmark [73], Finland [74] and Germany [75].

The second highest number (46) of studies (n = 110,641) was from Northern America.

Among the Northern American studies 40 were from the USA [15], [76]–[114], five from Canada [115]–[119] and one was from Mexico [120].

A total of 22 studies (n = 14,916) was identified in Asia, from 11 countries: eight were from Japan [121]–[128], three from India [129]–[131], two from each of South Korea [132], [133] and Thailand [134], [135], and one from each of Bangladesh [136], China [137], Hong Kong [138], Iran [139], Jordan [140], Taiwan [14], and Vietnam [141].

In total, 11 studies (n = 4,313) were reported from Latin America and Caribbean representing five countries. Among them, five were found from Argentina [142]–[146], three from Brazil [147]–[149], and one from each of Chile [150], Peru [151] and Venezuela [152].

Only four studies were identified from each of Africa (n = 626) and Oceania (n = 1,288) representing two and one countries, respectively. In Africa two studies were from each of Nigeria [153], [154] and South Africa [155], [156]. In Oceania, all the four studies were reported from Australia [157]–[160].

### Prevalence of *E. coli* O157 in cattle

At the global level the estimated prevalence of EcO157 in cattle ranged from 0.13% (95% CI, 0.04–0.33) [14] to 61.77% (95% CI, 56.63–66.71) [15] with substantial heterogeneity (*I* ^2^ = 98.7% P<0.001). The random effect estimated pooled prevalence at the global level was 5.68% (95% CI, 5.16–6.20). Overall and stratified pooled prevalence estimates of EcO157 in cattle by world region are presented in Table 2.

**Table 2:** Estimated pooled prevalence of *E. coli* O157 in cattle by world region.

World region	No. study	No. Cattle sampled	No. Positive cattle	Pooled estimate (%)	95% CI	Heterogeneity chi-squared (χ^2^)	*I^2^* (%)	P-value
**Global estimate**	140	220,427	12,683	5.68	5.16–6.20		98.7	<0.001
**Africa**	4	626	118	31.20	12.35–50.04	71.42	95.8	<0.001
**Asia**	22	14,916	937	4.69	3.05–6.33	1108.3	98.1	<0.001
**Europe**	53	88,643	5,425	5.15	4.21–6.09	3720.36	90.6	<0.001
**Latin America & Caribbean**	11	4,313	73	1.65	0.77–2.53	45.97	78.2	<0.001
**Northern America**	46	110,641	6,059	7.35	6.44–8.26	5187.73	99.1	<0.001
**Oceania**	4	1,288	71	6.85	2.41–11.29	41.15	92.7	0.002

**Figure 2:**
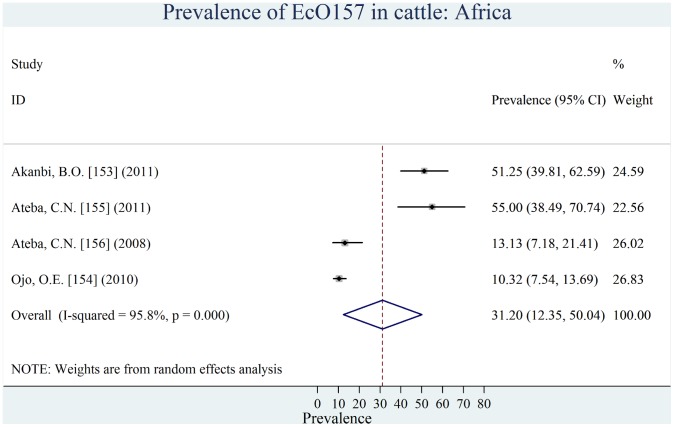
Forest plot of prevalence of *E. coli* O157 in cattle amongst studies conducted in Africa. (In all forest plots, the gray square around the dot represents the contribution of each study (weight) to the meta-analysis and the center dot represents point estimate).

**Figure 3:**
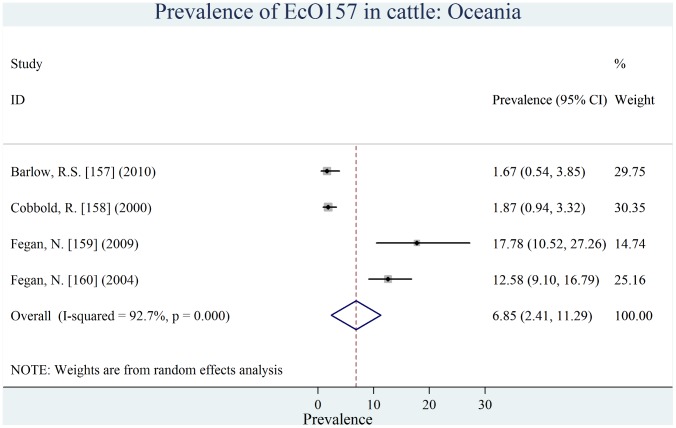
Forest plot of prevalence of *E. coli* O157 in cattle amongst studies conducted in Oceania.

**Figure 4:**
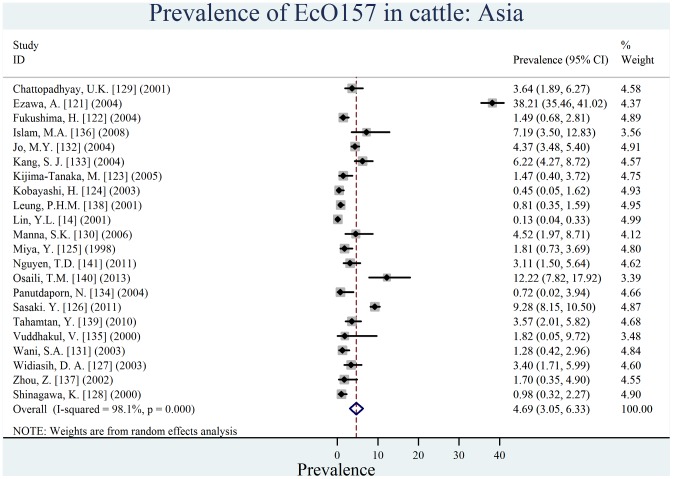
Forest plot of prevalence of *E. coli* O157 in cattle amongst studies conducted in Asia.

**Figure 5:**
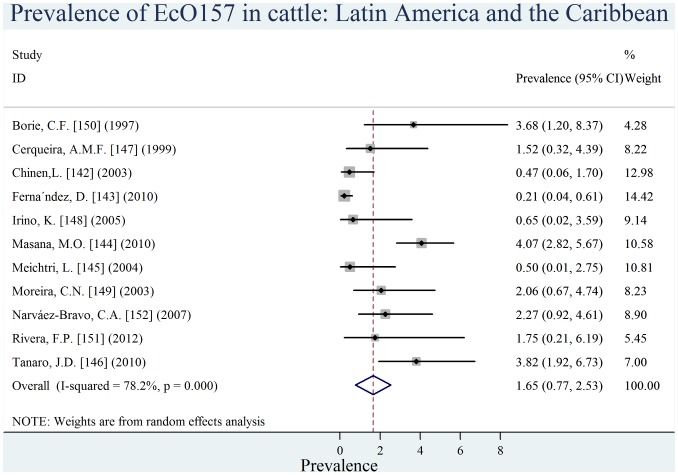
Forest plot of prevalence of *E. coli* O157 in cattle amongst studies conducted in Latin America and Caribbean.

**Figure 6:**
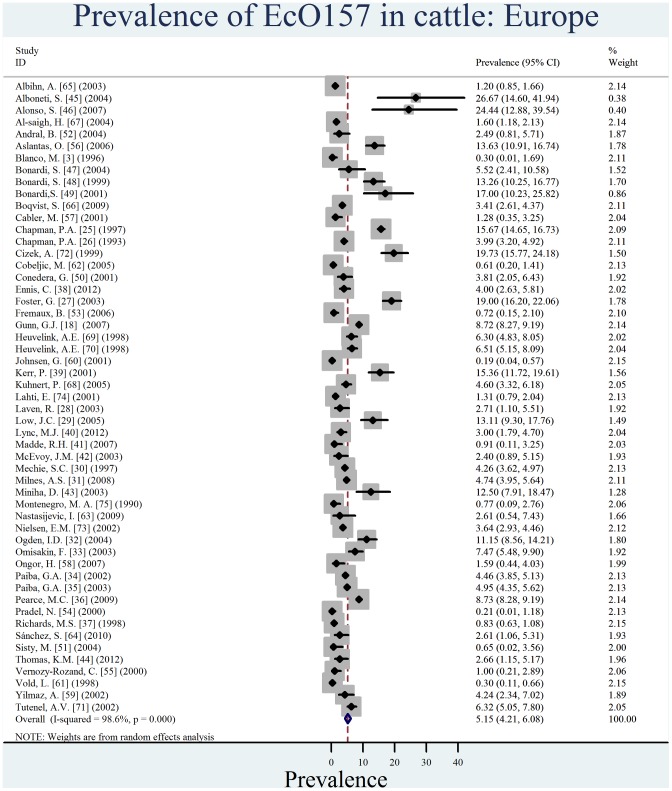
Forest plot of prevalence of *E. coli* O157 in cattle amongst studies conducted in Europe.

**Figure 7:**
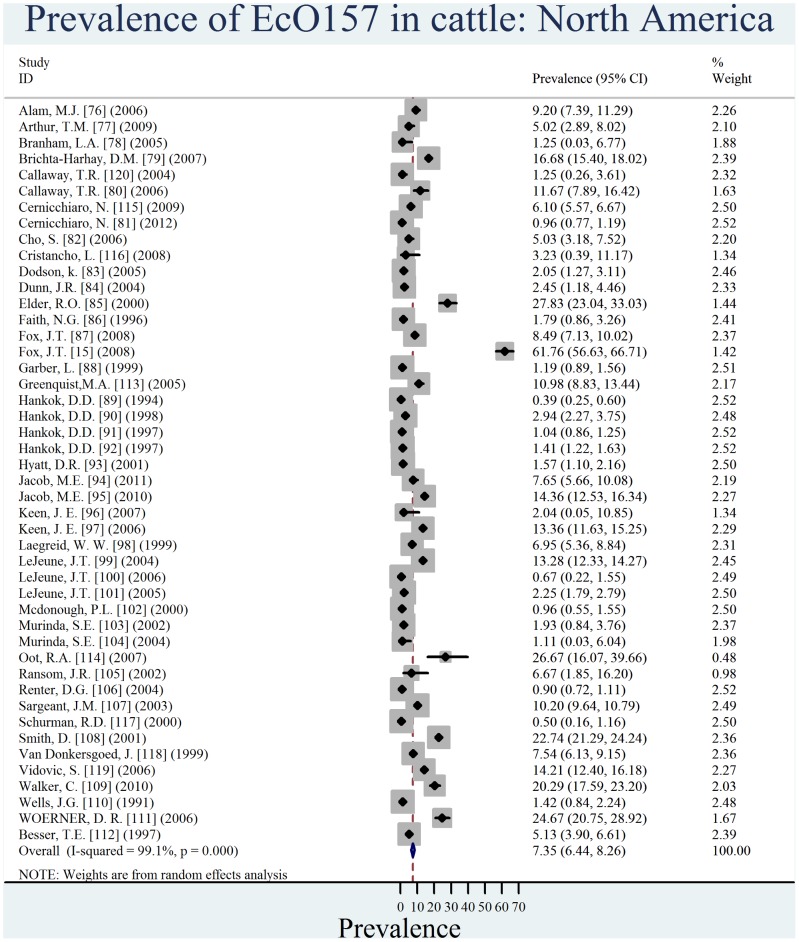
Forest plot of prevalence of *E. coli* O157 in cattle amongst studies conducted in North America.

Individual estimates of prevalence from contributing studies according to world region are outlined in Figures 2, 3, 4, 5, 6, 7. There was a wide regional variation in the prevalence of EcO157 in cattle, ranging from 1.65% (95% CI, 0.77–2.53) in Latin America and Caribbean to 31.20% (95% CI, 12.35–50.04) in Africa.

**Figure 8:**
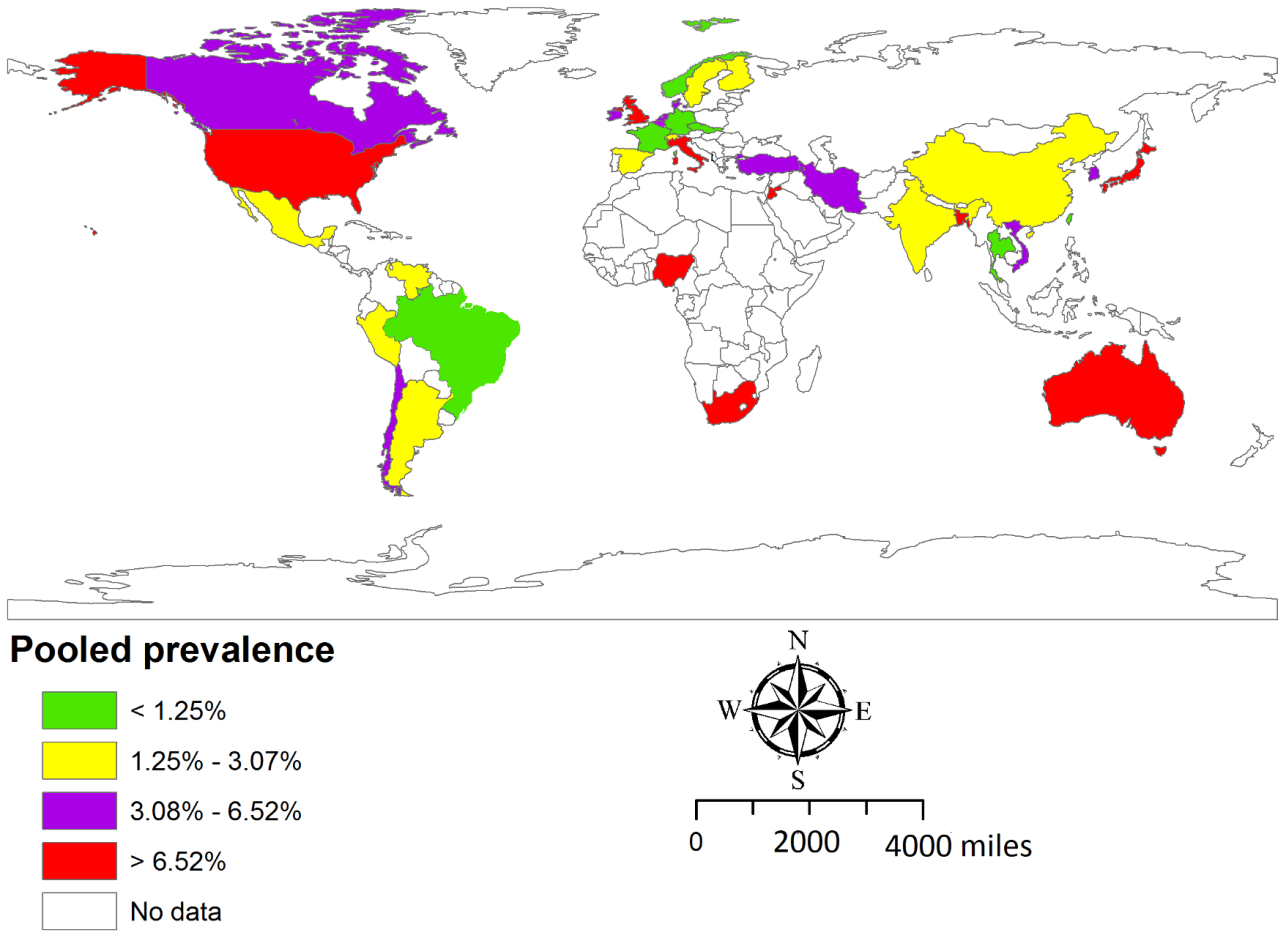
Estimated prevalence of *E. coli* O157 in cattle in different countries. The prevalence is based on a meta-analysis of 140 studies comprising 220,427 cattle from different production system. Regional adjusted prevalence is denoted by different colors and it represents the quartile distribution of prevalence by country.

The prevalence of EcO157 in cattle was also varied in countries of different world region. The estimates of adjusted prevalence of EcO157 in cattle in different countries are shown in Figure 8, by quartiles of prevalence.

### Sources of heterogeneity

In the prevalence of EcO157 in cattle four sources of heterogeneity were found in univariable meta-regression. They were world region (p<0.001), type of cattle (p<0.001), pre-enrichment (p = 0.027) and immunomagnetic separation (p = 0.024). The associations of the variables specimen (p = 0.066), health status (p = 0.080) and isolation media (p = 0.096) were borderline significant (Table 3). In the multivariable meta-regression model three variables world region (p<0.001), specimens (p = 0.074) and type of cattle (p<0.001) were found to be associated with the heterogeneity. An interaction term between ‘region’ and ‘type of cattle’ was added but it was not significant in the multivariable model.

**Table 3:** Meta-regression for prevalence of *E. coli* O157 in cattle.

Variables	Covariates	No. study (N-140)	Prevalence* (95%CI)	Univariable		Multivariable	
				Coef. (95%CI)	P-value	Coef. (95%CI)	P-value
**World region**					<0.001		<0.001
	**Asia (Ref.)**	22	4.69 (3.05, 6.33)				
	Africa	4	31.20 (12.35, 50.04)	23.17 (12.85, 33.49)	<0.001	22.37 (13.0, 31.75)	<0.001
	Europe	53	5.15 (4.21, 6.08)	0.97 (−3.34, 5.28)	0.658	−1.01 (−5.03, 3.01)	0.620
	Latin America and Caribbean	11	1.65 (0.77, 2.53)	−2.97 (−9.22, 3.28)	0.350	−3.67 (−9.64, 2.29)	0.225
	Northern America	46	7.35 (6.44, 8.26)	3.33 (−1.07, 7.73)	0.137	−0.24 (−4.57, 4.08)	0.911
	Oceania	4	6.85 (2.41, 11.29)	3.09 (−6.32, 12.50)	0.517	−0.99 (−9.56, 7.59)	0.820
**Specimens**					0.066		0.074
	**Rectal swab (Ref.)**	24	3.33 (2.24, 4.42)				
	Feces from rectum	55	6.67 (5.51, 7.82)	4.24 (−0.16, 8.63)	0.059	−0.02 (−4.06, 4.02)	0.992
	Feces from intestine	7	12.25 (4.48, 20.02)	8.72 (0.77, 16.67)	0.032	6.83 (−0.20, 13.87)	0.057
	Mixed type	13	8.77 (7.12, 10.41)	7.07 (0.87, 13.26)	0.026	3.91 (−1.72, 9.53)	0.171
	voided feces	41	5.39 (4.62, 6.18)	2.07 (−2.53, 6.67)	0.375	−0.95 (−5.13, 3.23)	0.653
**Origin of sampled cattle**					0.321		
	**Animal pen (Ref.)**	70	5.12 (4.45, 5.79)				
	Others	22	5.25 (3.27, 7.23)	−0.90 (−5.37, 3.57)	0.692		
	Slaughter house	48	7.10 (5.96, 8.24)	2.21 (−1.25, 5.67)	0.208		
**Type of cattle**					<0.001		<0.001
	**Dairy (Ref.)**	18	1.75 (1.26, 2.24)				
	Beef cattle	14	6.84 (4.03, 9.65)	4.96 (−0.77, 10.69)	0.089	2.07 (−3.65, 7.79)	0.476
	Others	96	4.85 (4.29, 5.41)	4.04 (−0.08, 8.17)	0.055	0.96 (−3.23, 5.16)	0.650
	Feedlot	12	19.58 (15.57, 23.59)	17.77 (11.65, 23.89)	<0.001	15.57 (9.54, 21.61)	<0.001
**Health status**					0.080		
	**Healthy (Ref.)**	18	2.62 (1.40, 3.84)				
	Not mentioned	113	6.55 (5.94, 7.16)	5.01 (0.44, 9.57)	0.032		
	Diseased	3	1.34 (0.34, 2.35)	−0.99 (−12.16, 10.18)	0.860		
	Mixed type	6	2.55 (0.81, 4.29)	.054(−8.41, 8.52)	0.990		
**Pre-enrichment**					0.027		0.110
	**With inhibitors (Ref.)**	80	7.82 (6.83, 8.80)				
	Others	9	5.64 (3.62, 7.66)	−1.12 (−7.49, 5.25)	0.729	4.79 (−2.50, 12.07)	0.195
	Without inhibitors	35	3.92 (3.04, 4.80)	−3.64 (−7.28, 0.01)	0.051	−3.15 (−5.33, 0.04)	0.053
	No pre-enrichment	15	1.42 (0.79, 2.06)	−6.91 (−11.92, −1.89)	0.007	−0.37 (−5.91, 5.17)	0.894
**IMS**					0.024		0.388
	**Yes (Ref.)**	90	7.67 (6.79, 8.54)				
	Others	6	1.41 (0.84, 1.97)	−5.09 (−12.60, 2.43)	0.183	−5.82 (−14.48, 2.83)	0.185
	No IMS	44	2.41 (1.89, 2.92)	−4.33 (−7.64, −1.02)	0.011	−1.16 (−4.85, 2.53)	0.535
**Isolation media**					0.096		0.289
	**CT-SMAC (Ref.)**	91	6.93 (6.24, 7.62)				
	CHROMagar O157	8	7.10 (2.76, 11.43)	−0.66 (−7.45, 6.13)	0.848	−4.03 (−9.91, 1.86)	0.178
	MacConkey	6	1.34 (0.42, 2.27)	−6.36 (−13.94, 1.22)	0.099	−1.72 (−8.99, 5.56)	0.641
	SMAC	13	2.22 (1.30, 3.15)	−5.17 (−10.51, 0.17)	0.058	−5.04 (−10.15, 0.07)	0.053
	Others	22	3.93 (2.39, 5.47)	−4.06 (−8.34, 0.22)	0.063	−2.50 (−6.70, 1.71)	0.242

*Estimated prevalence was calculated separately, Coef.  =  Regression coefficient, Ref.  =  Reference category, IMS = Immunomagnetic separation.

Studies in which the type of cattle was feedlot animal had significantly (p<0.001) higher prevalence of EcO157 compared with the studies that surveyed on other types of animal. It was evidenced in multivariable model that studies conducted in Africa had significantly (p<0.001) higher prevalence compared with other world regions.

## Discussion

This meta-analysis was based on a large number of cattle (220,427) derived from 140 studies representing 38 countries across the world, enabling us to assess reliable prevalence estimates of EcO157 at the global level.

To the best of our knowledge, this is the first meta-analysis of prevalence of EcO157 in cattle at the global level and the results indicate that the prevalence of EcO157 in cattle at the global level might be 5.68% (95% CI, 5.16–6.20), although the estimates varied ranging from 0.13% (95%CI, 0.04–0.33) [14] to 61.77% (95% CI, 56.63–66.71) [15]. The highest prevalence estimate (31.20%) was in African cattle and the estimates from each of the four studies from Africa [153]–[156] was comparably high, although each of two of them was based on the investigation of a sample size of only 120 cattle [153], [155].

On the other hand, a very low prevalence (1.65%) was from Latin America and Caribbean cattle [157]–[160]. A variable degree in prevalence was reported from Asia (4.69%), Europe (5.15%), Oceania (6.85%) and Northern America (7.35%). Compared with other countries in Asia, cattle in Jordan had the highest prevalence (12.22%, 95% CI, 7.82–17.92) [140] and the lowest (0.13%, 95% CI, 0.04–0.33) was estimated in Taiwan [14].

In Europe, the highest estimated prevalence was reported from Italy (10.45%, 95% CI, 5.30–15.61) [45]–[51] and the lowest from Norway (0.25%, 95% CI, 0.06–0.42) [60], [61].

Northern America was well represented in this study with 40 studies from the USA and the prevalence estimate was higher (7.60%) in this country compared with Canada and Mexico. The diverse prevalence estimates of EcO157 in cattle among the studies of different world regions might be due to reflections of geographical variations, or attributable to underlying risk factors.

Strong evidence was found leading to the variation in the prevalence of EcO157 in cattle. Meta-regression model enabled us to evaluate the impact of both methodological differences and some other factors on the prevalence estimates of EcO157 in cattle. About 46% between study heterogeneity was explained by the final multivariable model, indicating that other biological and/or methodological factors are responsible for the remaining between study variance. The residual heterogeneity was 98%. The Joint test for all covariates (P<0.001) also showed evidence for an association of covariates with the prevalence of EcO157. The interaction between variables ‘world region’ and ‘type of cattle’ was included as an interaction term in multivariable meta-regression model but this interaction term was found non-significant (p>0.20). The study revealed that type of cattle (dairy/beef/feedlot/others) plays a vital role in the variation of regional prevalence (Table 3). This finding is supported by Jeon et al. [161] who stated that the level of EcO157 carriage in cattle is influenced by many animal related factors. In this study the prevalence of EcO157 was estimated to be 19.58% (95% CI, 15.57–23.59) in feedlot cattle, substantially higher to the level (1.75%; 95% CI, 1.26–2.24) estimated in dairy cattle. The types of specimen collected from cattle were also responsible for the variability in the estimates. There were several types of specimen used to isolate the organism namely rectal swab, feces from intestine, feces from rectum, voided feces and mixed types. When feces were collected directly from the intestine then the estimates were higher compared to other types of specimen investigated. On the other hand, diversity in the methodological steps followed in the isolation of the organism, especially pre-enrichment and type of isolation media was found responsible for the global variation in the prevalence of EcO157 in cattle. In relation to the methods of pre-enrichment, the highest estimated prevalence (7.82%) was found in the studies in which specific inhibitors were used compared to other pre-enrichment methods. In addition, the amount of specimen matrix, particularly feces could be an important factor to influence the detection rate of EcO157 in cattle [79]. This factor might also have a role in the sources of between study heterogeneity, but we couldn't include it (amount of feces) in this meta-analysis because not feces but rectal swabs or swabs plus feces, in which the amounts of investigated matrix could not be estimated, were also the primary samples used in many studies. Similarly, a higher level of prevalence (7.67%) was reported where IMS techniques were employed. Season is a potential explanatory variable in the prevalence of EcO157 in cattle [32], [45], [65], [76], [77]. This was also not included in this study because of the limitation of data sources and different seasonal parameters used in different studies.

However, the outputs of meta-regression need to be explained with caution. Some covariates were reported in a few studies, for example, the covariate ‘disease’ was found only in three studies [37], [131], [141], shrinking the association detection power of meta-regression. Furthermore, some categories were explored as sources of heterogeneity between studies but were classified as ‘others’ or ‘unknown’, thereby failing to give sufficient information. Residual confounding is also an important issue to consider when dealing with observational studies.

**Figure 9:**
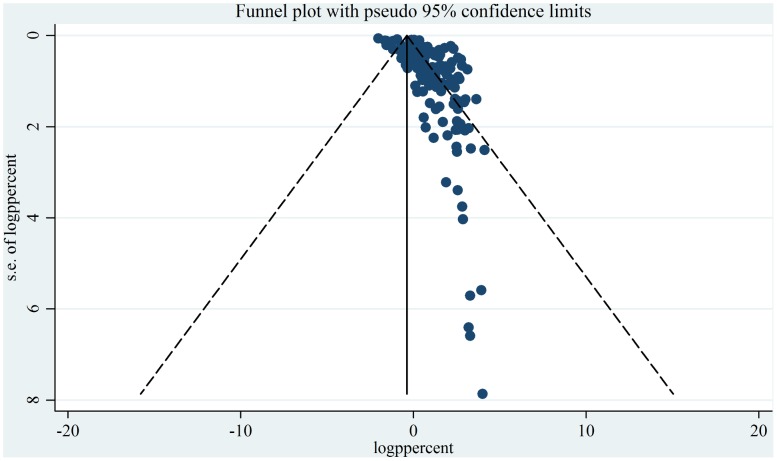
Funnel plot for examination of publication bias. (ppercent, prevalence percent; se, standard error).

The extent of publication bias in the selected studies was measured and demonstrated by the funnel plot (Figure 9). It is clearly not symmetrical and some of the points fall outside of the funnel, indicating publication bias. The sources of the funnel plot asymmetry were tested by Egger test [162], the result of which confirmed the small study effects. The estimated bias co-efficient was 6.79 with a standard error of 0.084 providing a p-value of <0.001. Thus the test demonstrates strong evidence to the presence of small study effects. However, there are many different possible factors for funnel plot asymmetry, namely selection bias, true heterogeneity, data irregularities, artifact as well as by chance [162].

There are some limitations in this study. Firstly, there were a very few reports from some world regions, especially Africa and Oceania. Thus a true reflection of the prevalence from these regions could not be obtained. Furthermore, all the four studies from Oceania are in fact from Australia [157]–[160] and out of the 53 countries in Africa only two: Nigeria [153], [154] and South Africa [155], [156] are represented in this study with two studies from each of them.

Secondly, most of the studies drew samples from large commercial cattle production system and none of the studies reported any prevalence in backyard or smallholdings' cattle population which occupy a large proportion of the total cattle population in the developing world, especially in Asia and Africa.

Thirdly, unpublished studies, conference abstract, experimental and intervention studies were not included in this meta-analysis though it could bring some more publications. Besides, the authors of this study could not find 103 full text papers even after contacting the corresponding authors of all these studies. Additionally, some other potential factors like, age dependent factors and seasonal pattern in the prevalence are to be included in the study to explore their effects on the regional estimates. Due to unavailability of data these factors could not be evaluated in this study. Finally, a significant heterogeneity between studies was detected even within a particular region.

In conclusion, the prevalence of EcO157 in cattle at the global level seems to be 5.68%. The random effects pooled prevalence estimates of it in Africa, Northern America, Oceania, Europe, Asia and Latin America-Caribbean are likely to be 31.20%, 7.35%, 6.85%, 5.15%, 4.69% and 1.65%, respectively, although between studies heterogeneity was evidenced in most of these world regions. Excluding the regional, other factors that might have roles in the variation of EcO157 prevalence estimates include kind of screening specimen matrix, type of cattle, and probably method of pre-enrichment applied for the organism. Their precise roles in varying the detection level of the organism need to be investigated in laboratory to suggest a better methodology to undertake any prevalence study for EcO157 in future.

## Acknowledgments

The authors would like to acknowledge WHO for HINARI Program which have enabled developing countries to gain access to biomedical literature database. They would like to thank Himel Barua to locate some full text articles. They would like to thank many other authors who supplied their paper by email.

## Supporting Information

Checklist S1 PRISMA checklist.
(DOC)

File S1 A pretested data extraction spreadsheet.
(XLS)

File S2 Description of studies reporting prevalence of *E. coli* O157 in cattle.
(DOC)

File S3 List of papers included in this meta-analysis.
(DOC)

File S4 List of excluded full text paper with proper justification.
(DOC)
