# Supplementary material for: Regional Variation in the Prevalence of E. coli O157 in Cattle: A Meta-Analysis and Meta-Regression
Source: PLoS One. 2014 Apr 1;9(4):e93299. doi: 10.1371/journal.pone.0093299 (PMC3972218; doi:10.1371/journal.pone.0093299)
Supplement: File S2 — Description of studies reporting prevalence of E. coli O157 in cattle. (DOC) [file pone.0093299.s003.doc]

Supplementary file 2: Description of studies reporting prevalence of *E. coli* O157 in cattle throughout the world

| **First Author** | **Year of publication** | **Country** | **Specimen (s)** | **No. Sample** | **No. Positive** |
| --- | --- | --- | --- | --- | --- |
| Akanbi, B.O. | 2011 | Nigeria | Feces from colorectal | 80 | 41 |
| Alam, M.J. | 2006 | USA | Feces (fresh) | 891 | 82 |
| Albihn, A. | 2003 | Sweden | Feces from rectum | 3071 | 37 |
| Alboneti, S. | 2004 | Italy | Feces from rectum | 45 | 12 |
| Alonso, S. | 2007 | Italy | Feces from colon | 45 | 11 |
| Al-saigh, H. | 2004 | Switzerland | Feces (site not mentioned) | 2 930 | 47 |
| Andral, B. | 2004 | France | Feces | 201 | 5 |
| Arthur, T.M. | 2009 | USA | Feces from rectum | 319 | 16 |
| Aslantas, O. | 2006 | Turkey | Rectal swabs | 565 | 77 |
| Ateba, C.N. | 2011 | South Africa | Feces from rectum | 40 | 22 |
| Ateba, C.N. | 2008 | South Africa | Feces from rectum | 99 | 13 |
| Barlow, R.S. | 2010 | Australia | feces from proximal rectum | 300 | 5 |
| Blanco, M. | 1996 | Spain | fecal swab | 328 | 1 |
| Bonardi, S. | 2004 | Italy | feces from caecum | 145 | 8 |
| Bonardi, S. | 1999 | Italy | feces from rectum | 445 | 59 |
| Bonardi,S. | 2001 | Italy | feces from rectum | 100 | 17 |
| Boqvist, S. | 2009 | Sweden | feces from ampulla recti | 1758 | 60 |
| Borie, C.F., | 1997 | Chile | Rectal swabs | 136 | 5 |
| Branham, L.A. | 2005 | USA | Feces from rectum | 80 | 1 |
| Brichta-Harhay, D.M. | 2007 | USA | Fecal grab samples | 3190 | 532 |
| Cabler, M. | 2001 | Turkey | Feces from rectum | 312 | 4 |
| Callaway, T.R. | 2004 | Mexico | Feces from rectum | 240 | 3 |
| Callaway, T.R. | 2006 | USA | fecal pat from pan | 240 | 28 |
| Cernicchiaro, N. | 2009 | Canada | fecal pat from pan | 7519 | 459 |
| Cernicchiaro, N. | 2012 | USA | fecal pat from pan | 8940 | 86 |
| Cerqueira, A.M.F. | 1999 | Brazil | Rectal swabs | 197 | 3 |
| Chapman, P.A. | 1997 | UK | rectal swab | 4800 | 752 |
| Chapman, P.A. | 1993 | UK | rectal swab | 2103 | 84 |
| Chattopadhyay, U.K. | 2001 | India | feces from rectum | 330 | 12 |
| Chinen, J. | 2003 | Argentina | fecal swab | 422 | 2 |
| Cho, S. | 2006 | USA | Feces from rectum | 437 | 22 |
| Cizek, A. | 1999 | Czech republic | mixed type | 365 | 72 |
| Cobbold, R. | 2000 | Australia | feces from rectum | 588 | 11 |
| Cobeljic, M. | 2005 | Serbia | mixed type | 824 | 5 |
| Conedera, G. | 2001 | Italy | feces by rectal swab | 341 | 13 |
| Cristancho, L. | 2008 | Canada | feces from rectum | 62 | 2 |
| Dodson, k. | 2005 | USA | feces from rectum | 1026 | 21 |
| Dunn, J.R. | 2004 | USA | feces from rectum | 408 | 10 |
| Elder, R.O. | 2000 | USA | feces from colon | 327 | 91 |
| Ennis, C. | 2012 | Ireland | freshly voided feces | 650 | 26 |
| Ezawa, A. | 2004 | Japan | feces from rectum | 1209 | 462 |
| Faith, N.G. | 1996 | USA | feces from rectum | 560 | 10 |
| Fegan, N. | 2009 | Australia | feces from rectum | 90 | 16 |
| Fegan, N. | 2004 | Australia | feces from intestine | 310 | 39 |
| Ferna´ndez, D. | 2010 | Argentina | rectal swab | 1440 | 3 |
| Foster, G. | 2003 | UK | fresh fecal pats | 721 | 137 |
| Fox, J.T. | 2008 | USA | feces from rectum | 1495 | 127 |
| Fox, J.T. | 2008 | USA | mixed type | 374 | 231 |
| Fremaux, B. | 2006 | French | feces from cattle | 415 | 3 |
| Fukushima, H. | 2004 | Japan | rectal swab immediately | 605 | 9 |
| Garber, L. | 1999 | USA | feces from rectal retrieval | 4361 | 52 |
| Greenquist,M.A. | 2005 | USA | mixed type | 747 | 82 |
| Gunn, G.J. | 2007 | Scotland UK | fresh fecal pat sample | 14856 | 1296 |
| Hankok, D.D | 1994 | USA | mixed type | 5582 | 22 |
| Hankok, D.D | 1998 | USA | fecal pat samples | 2143 | 63 |
| Hankok, D.D | 1997 | USA | mixed type | 10832 | 113 |
| Hankok, D.D | 1997 | USA | fecal pat | 12664 | 179 |
| Heuvelink, A.E. | 1998 | The Netherlands | feces from rectum | 937 | 59 |
| Heuvelink, A.E. | 1998 | The Netherlands | feces from rectum | 1152 | 75 |
| Hyatt, D.R. | 2001 | USA | fecal pat samples | 2297 | 36 |
| Irino, K. | 2005 | Brazil | rectal swab | 153 | 1 |
| Islam, M.A. | 2008 | Bangladesh | feces from rectum | 139 | 10 |
| Jacob, M.E. | 2011 | USA | mixed type | 601 | 46 |
| Jacob, M.E. | 2010 | USA | feces from rectum | 1351 | 194 |
| Jo, M.Y. | 2004 | Korea | fecal sample | 1854 | 81 |
| Johnsen, G | 2001 | Norway | feces from intestine | 1541 | 3 |
| Kang, S. J. | 2004 | Republic of Korea | fecal specimens | 498 | 31 |
| Keen, J. E. | 2007 | USA | mixed type | 49 | 1 |
| Keen, J. E. | 2006 | USA | fresh feces | 1407 | 188 |
| Kerr, P. | 2001 | Ireland | fresh feces | 345 | 53 |
| Kijima-Tanaka, M. | 2005 | Japan | fresh fecal sample | 272 | 4 |
| Kobayashi, H. | 2003 | Japan | rectal stool grab | 444 | 2 |
| Kuhnert, P. | 2005 | Switzerland | feces from rectum | 892 | 41 |
| Laegreid, W. W. | 1999 | USA | rectal grab | 878 | 61 |
| Lahti, E. | 2001 | Finland | Rectal fecal samples | 1448 | 19 |
| Laven, R. | 2003 | UK | feces from rectum | 258 | 7 |
| LeJeune, J.T. | 2004 | USA | fecal pats | 4790 | 636 |
| LeJeune, J.T. | 2006 | USA | recto-anal mucosal swab | 750 | 5 |
| LeJeune, J.T. | 2005 | USA | fecal pats | 3600 | 81 |
| Leung, P.H.M. | 2001 | Hong Kong | rectal swab | 986 | 8 |
| Lin, Y.L. | 2001 | Taiwan | feces from rectum by swab | 3062 | 4 |
| Low, J.C. | 2005 | Scotland | feces from RAJ | 267 | 35 |
| Lync, M.J. | 2012 | Ireland | rectal fecal swabs | 600 | 18 |
| Madde, R.H. | 2007 | Ireland | feces from rectum | 220 | 2 |
| Manna, S.K. | 2006 | India | mixed type | 177 | 8 |
| Masana, M.O. | 2010 | Argentina | feces from rectum | 811 | 33 |
| Mcdonough, P.L. | 2000 | USA | Bovine fecal specimens | 1668 | 16 |
| McEvoy, J.M. | 2003 | Ireland | feces from rectum | 250 | 6 |
| Mechie, S.C. | 1997 | UK | rectal swab | 3593 | 153 |
| Meichtri, L. | 2004 | Argentina | mixed type | 200 | 1 |
| Milnes, A.S. | 2008 | Great Britain | rectal contents | 2553 | 121 |
| Miniha, D. | 2003 | Ireland | rectal fecal samples | 168 | 21 |
| Miya, Y. | 1998 | Japan | feces or rectal contents | 387 | 7 |
| Montenegro,M. A. | 1990 | Germany | Fecal samples | 259 | 2 |
| Moreira, C.N. | 2003 | Brazil | swabs of rectal feces | 243 | 5 |
| Murinda, S.E. | 2002 | USA | feces from rectum | 415 | 8 |
| Murinda, S.E. | 2004 | USA | fecal swab | 90 | 1 |
| Narváez, B.C.A. | 2007 | Venezuela | fecal samples | 309 | 7 |
| Nastasijevic, I. | 2009 | Serbia | feces from terminal rectum | 115 | 3 |
| Nguyen, T.D. | 2011 | Vietnam | rectal swabs | 322 | 10 |
| Nielsen, E.M. | 2002 | Denmark | mixed type | 2419 | 88 |
| Ogden, I.D. | 2004 | Scotland | feces by rectal retrieval | 511 | 57 |
| Ojo, O.E. | 2010 | Nigeria | feces from rectum | 407 | 42 |
| Omisakin, F. | 2003 | Scotland | feces from rectum | 589 | 44 |
| Ongor, H. | 2007 | Turkey | Rectal swab | 251 | 4 |
| Oot, R.A. | 2007 | USA | feces | 60 | 16 |
| Osaili, T.M. | 2013 | Jordan | feces from colon | 180 | 22 |
| Paiba, G.A. | 2002 | Great Britain | feces from rectum | 4173 | 186 |
| Paiba, G.A. | 2003 | England and Wales | feces from rectum | 4663 | 231 |
| Panutdaporn, N. | 2004 | Thailand | fecal samples | 139 | 1 |
| Pearce, M.C. | 2009 | Scotland | fecal pats | 14849 | 1296 |
| Pradel, N. | 2000 | France | feces sample | 471 | 1 |
| Ransom, J.R. | 2002 | USA | mixed type | 60 | 4 |
| Renter, D.G. | 2004 | USA | Freshly voided feces | 9122 | 82 |
| Richards, M.S. | 1998 | England and Wales | feces sample | 6495 | 54 |
| Rivera, F.P. | 2012 | Peru | swabs of rectal feces | 114 | 2 |
| Sánchez, S. | 2010 | Spain | Rectal–anal swabs | 268 | 7 |
| Sargeant, J.M. | 2003 | USA | Freshly voided feces | 10662 | 1088 |
| Sasaki. Y. | 2011 | Japan | rectal content grab sample | 2436 | 226 |
| Schurman, R.D. | 2000 | Canada | fecal swabs | 1000 | 5 |
| Sisty, M. | 2004 | Italy | feces from rectum | 154 | 1 |
| Smith, D. | 2001 | USA | feces from rectum | 3162 | 719 |
| Tahamtan, Y. | 2010 | Iran | recto-anal mucosal swabs | 420 | 15 |
| Tanaro, J.D. | 2010 | Argentina | swabs of rectal feces | 288 | 11 |
| Thomas, K.M. | 2012 | Ireland | feces from rectum | 301 | 8 |
| Van Donkersgoed, J. | 1999 | Canada | feces from rectum | 1247 | 94 |
| Vernozy-Rozand, C. | 2000 | France | fecal samples | 300 | 3 |
| Vidovic, S. | 2006 | Canada | freshly defecated feces | 1365 | 194 |
| Vold, L. | 1998 | Norway | feces from rectum | 1970 | 6 |
| Vuddhakul, V | 2000 | Thailand | fresh feces | 55 | 1 |
| Walker, C. | 2010 | USA | gut contents | 823 | 167 |
| Wani, S.A. | 2003 | India | feces from rectum | 391 | 5 |
| Wells, J.G. | 1991 | USA | Swabs from stool | 1266 | 18 |
| Widiasih, D. A. | 2003 | Japan | rectal stool grab samples | 324 | 11 |
| WOERNER, D. R. | 2006 | USA | inside of fresh fecal pats | 450 | 111 |
| Yilmaz, A. | 2002 | Turkey | rectal feces by swab | 330 | 14 |
| Zhou, Z. | 2002 | China | feces by swab | 176 | 3 |
| Tutenel, A.V. | 2002 | Belgium | fecal sample | 1281 | 81 |
| Besser, T.E. | 1997 | USA | rectal swab samples | 1091 | 56 |
| Shinagawa, K. | 2000 | Japan | fecal specimens | 510 | 5 |
