# Supplementary material for: Regional Variation in the Prevalence of E. coli O157 in Cattle: A Meta-Analysis and Meta-Regression
Source: PLoS One. 2014 Apr 1;9(4):e93299. doi: 10.1371/journal.pone.0093299 (PMC3972218; doi:10.1371/journal.pone.0093299)
Supplement: File S3 — List of papers included in this meta-analysis. (DOC) [file pone.0093299.s004.doc]

**Articles included in this study (n=140)**

1. Akanbi BO, Mbah IP, Kerry PC (2011) "Prevalence of *Escherichia coli* O157:H7 on hides and faeces of ruminants at slaughter in two major abattoirs in Nigeria." Lett Appl Microbiol 53(3): 336-340.
2. Alam M J, Zurek L (2006) "Seasonal prevalence of *Escherichia coli* O157:H7 in beef cattle feces." J Food Prot 69(12): 3018-3020.
3. Albihn A, Eriksson E, Wallen C, Aspán A (2003) "Verotoxinogenic *Escherichia coli* (VTEC) O157:H7--a nationwide Swedish survey of bovine faeces." Acta Vet Scand 44(1-2): 43-52.
4. Albonetti S, Trevisani M, Alonso-Alveraz S, Rosmini R (2004) "Detection of *Escherichia coli*  serotypes 0157 in beef carcasses and faecal material." Vet Res Commun 28 Suppl 1: 249-251.
5. Alonso S, Azucena M, Blanco M, Blanco JE, Dahbi G, Ferreiro MT, López C, Alberghini L, Albonetti S, Echeita A,Trevisani M, Blanco J (2007) "Fecal carriage of *Escherichia coli*  O157:H7 and carcass contamination in cattle at slaughter in northern Italy." Int Microbiol 10(2): 109-116.
6. Al-Saigh H, Zweifel C, Blanco J, Blanco JE, Blanco M, Usera MA, Stephan R (2004) "Fecal shedding of *Escherichia coli*  O157, Salmonella, and Campylobacter in Swiss cattle at slaughter." J Food Prot 67(4): 679-684.
7. Andral B, Aspan A, Perelle S, Fach P (2004) "PCR detection of virulence genes and molecular epidemiology of STEC O157 isolates from French abattoirs." Vet Rec 155(12): 365-368.
8. Arthur TM, Keen JE, Bosilevac JM, Brichta-Harhay DM, Kalchayanand N, Shackelford SD, Wheeler TL, Nou X, Koohmaraie M (2009) "Longitudinal study of *Escherichia coli*  O157:H7 in a beef cattle feedlot and role of high-level shedders in hide contamination." Appl Environ Microbiol 75(20): 6515-6523.
9. Aslantas Ö, Erdog S, Cantekin Z, Gulacti I, Gulsum A. Evrendilek (2006) "Isolation and characterization of verocytotoxin-producing *Escherichia coli*  O157 from Turkish cattle." Int J Food Microbiol 106(3): 338-342.
10. Ateba CN, Mbewe M (2011) "Detection of *Escherichia coli* O157:H7 virulence genes in isolates from beef, pork, water, human and animal species in the northwest province, South Africa: public health implications." Res Microbiol 162(3): 240-248.
11. Ateba CN, Mbewe M, Bezuidenhout CC (2008) "Prevalence of *Escherichia coli* O157 strains in cattle, pigs and humans in North West province, South Africa." South African Journal of Science 104(1-2): 7-8.
12. Barlow RS, Mellor GE (2010) Prevalence of enterohemorrhagic *Escherichia coli*  serotypes in Australian beef cattle. Foodborne pathogens and disease Volume 7, Number 10, 1239-1245.
13. Besser TE, Hancock DD, Pritchett LC, McRae EM, Rice DH, Tarr PI (1997) Duration of detection of fecal excretion of *Escherichia coli*  O157:H7 in cattle. J. Infect. Dis. 175, 726–729.
14. Blanco M, Blanco JE, Blanco J, Gonzalez ea, Mora A, Prado C, Fernandez L, Rio M, Ramos J, Alonso MP (1996) "Prevalence and characteristics of *Escherichia coli*  serotype O157:H7 and other verotoxin-producing E-coli in healthy cattle." Epidemiol Infect 117(2): 251-257.
15. Bonardi S, Foni E, Brindani F, Bacci C, Chiapponi C, Cavallini P (2004) "Detection and characterization of verocytotoxin-producing *Escherichia coli*  (vtec) O157 and non-O157 in cattle at slaughter." New Microbiol 27(3): 255-261.
16. Bonardi S, E. Bottarellia MA, Pacciarinib ML, Ansuinib A, Vellinic G, Morabitod S, Caprioliet A (1999). "Isolation of Verocytotoxin-producing *Escherichia coli* O157:H7 from cattle at slaughter in Italy." Vet Microbiol 67(3): 203-211.
17. Bonardi S, Maggi E, Pizzin G, Morabito S, Caprioli A (2001) "Faecal carriage of Verocytotoxin-producing *Escherichia coli*  O157 and carcass contamination in cattle at slaughter in northern Italy." Int J Food Microbiol 66(1-2): 47-53.
18. Boqvist S, Aspan A, Eriksson E (2009) "Prevalence of verotoxigenic *Escherichia coli* O157:H7 in fecal and ear samples from slaughtered cattle in Sweden." J Food Prot 72(8): 1709-1712.
19. Borif CF, Monreal Z, Martinez J, Arellano C, Prado V (1997) "Detection and characterization of enterohaemorrhagic *Escherichia coli* in slaughtered cattle." Zentralbl Veterinarmed B 44(5): 273-279.
20. Branham LA, Carr MA, Scott CB, Callaway TR (2005) "*E. coli* O157 and Salmonella spp. in white-tailed deer and livestock." Curr Issues Intest Microbiol 6(2): 25-29.
21. Brichta-Harhay DM, Arthur TM, Bosilevac JM, Guerini MN, Kalchayanand N, Koohmaraie M (2007) "Enumeration of Salmonella and *Escherichia coli*  O157:H7 in ground beef, cattle carcass, hide and faecal samples using direct plating methods." J Appl Microbiol 103(5): 1657-1668.
22. Cabalar M, Boynukara B, Gulhan T, Hakký ekin I (2001) "Prevalence of rotavirus, *Escherichia coli*  K99 and O157 : H7 in healthy dairy cattle herds in Van, Turkey." Turk J Vet Anim Sci 25(2): 191-196.
23. Callaway TR, Anderson RC, Tellez G, Rosario C, Nava GM, Eslava C, Blanco MA, Quiroz MA, Olgui´n A, Herradora M, Edrington TS, Genovese KJ, Harvey RB, Nisbet DJ (2004) "Prevalence of *Escherichia coli*  O157 in cattle and swine in central Mexico." J Food Prot 67(10): 2274-2276.
24. Callaway TR, Edrington TS, Brabban AD, Keen JE, Anderson RC, Rossman ML, Engler MJ, Genovese KJ, Gwartney BL, Reagan JO, Poole TL, Harvey RB, Kutter EM, Nisbet DJ (2006). "Fecal prevalence of *Escherichia coli*  O157, Salmonella, Listeria, and Bacteriophage Infecting *E. coli* O157:H7 in feedlot cattle in the Southern Plains region of the United States." Foodborne Pathog Dis 3(3): 234-244.
25. Cernicchiaro N, Pearl DL, Ghimire S, Gyles CL, Johnson RP, LeJeune JT, Ziebell K, McEwen SA (2009). "Risk factors associated with *Escherichia coli* O157:H7 in Ontario beef cow-calf operations." Prev Vet Med 92(1-2): 106-115.
26. Cernicchiaro N, Pearl DL, McEwen SA, Harpster L, Homan HJ, Linz GM, LeJeune JT (2012) "Association of wild bird density and farm management factors with the prevalence of *E. coli* O157 in dairy herds in Ohio (2007-2009)." Zoonoses Public Health 59(5): 320-329.
27. Cerqueira AMF, Guth BEC, Joaquim RM, Andrade JRC (1999) "High occurrence of Shiga toxin-producing *Escherichia coli*  (STEC) in healthy cattle in Rio de Janeiro State, Brazil." Vet Microbiol 70(1-2): 111-121.
28. Chapman PA, Siddons CA, Malo ATC, Harkin MA (1997) "A 1-year study of *Escherichia coli* O157 in cattle, sheep, pigs and poultry." Epidemiol Infect 119(2): 245-250.
29. Chapman PA, Siddons CA, Wright DJ, Norman P, Fox J, Crick E (1993) "Cattle as a possible source of verocytotoxin-producing *Escherichia coli*  O157 infections in man." Epidemiol Infect 111(3): 439-447.
30. Chattopadhyay UK, Dutta S, Deb A. Pal D (2001) "Verotoxin-producing *Escherichia coli* --an environment-induced emerging zoonosis in and around Calcutta." Int J Environ Health Res 11(1): 107-112.
31. Chinen I, Otero JL, Miliwebsky ES, Rold ML, Baschkier A, Chillemi GM, Oboli CN, Frizzo L, Rivas M (2003) "Isolation and characterisation of Shiga toxin-producing *Escherichia coli*  O157:H7 from calves in Argentina." Res Vet Sci 74(3): 283-286.
32. Cho S, Bender JB, Diez-gonzalez F, Fossler CP, Hedberg CW, Kaneene JB, Ruegg PL, Warnick LD, Wells SJ (2006) "Prevalence and characterization of *Escherichia coli*  O157 isolates from Minnesota dairy farms and county fairs." J Food Prot 69(2): 252-259.
33. Cizek A, Alexa P, Literak I, Hamrik J, Novak P, Smola J (1999) "Shiga toxin-producing *Escherichia coli*  O157 in feedlot cattle and Norwegian rats from a large-scale farm." Lett Appl Microbiol 28(6): 435-439.
34. Cobbold R, Desmarchelier P (2000) "A longitudinal study of Shiga-toxigenic *Escherichia coli*  (STEC) prevalence in three Australian dairy herds." Vet Microbiol 71(1-2): 125-137.
35. Cobeljic M, Dimic B, Opacic D, Lepsanovic Z, Stojanovic V, Lazic S(2005) "The prevalence of Shiga toxin-producing *Escherichia coli*  in domestic animals and food in Serbia." Epidemiol Infect 133(2): 359-366.
36. Conedera G, Chapman PA, Marangon S, Tisato E, Dalvit P, Zuinet A (2001) "A field survey of *Escherichia coli* O157 ecology on a cattle farm in Italy." Int J Food Microbiol 66(1-2): 85-93.
37. Cristancho L, Johnson RP, McEwen SA, Gyles CL (2008) "*Escherichia coli* O157:H7 and other Shiga toxin-producing *E. coli* in white veal calves." Vet Microbiol 126(1-3): 200-209.
38. Dodson K, LeJeune J (2005) "*Escherichia coli* O157:H7, Campylobacter jejuni, and Salmonella Prevalence in cull dairy cows marketed in northeastern Ohio." J Food Prot 68(5): 927-931.
39. Dunn JR, Keen JE, Vecchio RD, Wittum TE, Thompson RA (2004) "*Escherichia coli*  O157:H7 in a cohort of weaned, preconditioned range beef calves." J Food Prot 67(11): 2391-2396.
40. Elder RO, Keen JE, Siragusa GR, Barkocy-Gallagher GA, Koohmaraie M, Laegreid WW (2000) "Correlation of enterohemorrhagic *Escherichia coli*  O157 prevalence in feces, hides, and carcasses of beef cattle during processing." Proc Natl Acad Sci U S A 97(7): 2999-3003.
41. Ennis C, McDowell D, Bolton DJ (2012) "The prevalence, distribution and characterization of Shiga toxin-producing *Escherichia coli* (STEC) serotypes and virulotypes from a cluster of bovine farms." J Appl Microbiol 113(5): 1238-1248.
42. Ezawa A, Gocho F, Saitoh M, Tamura T, Kawata K, Takahashi T, Kikuchi N (2004) "A three-year study of enterohemorrhagic *Escherichia coli* O157 on a farm in Japan." J Vet Med Sci 66(7): 779-784.
43. Faith NG, Shere JA, Brosch R, Arnold KW, Ansay SE, Lee MS, Luchansky JB, Kaspar CW (1996) "Prevalence and clonal nature of *Escherichia coli*  O157:H7 isolated on dairy farms in Wisconsin." Abstracts of the General Meeting of the American Society for Microbiology 95(0): 384.
44. Fegan N, Higgs G, Duffy LL, Barlow RS (2009) "The effects of transport and lairage on counts of *Escherichia coli* O157 in the feces and on the hides of individual cattle." Foodborne Pathog Dis 6(9): 1113-1120.
45. Fegan N, Vanderlinde P, Higgs G, Desmarchelier P (2004) "The prevalence and concentration of *Escherichia coli* O157 in faeces of cattle from different production systems at slaughter." J Appl Microbiol 97(2): 362-370.
46. Fernandez D, Irino K, Sanz ME, Padola NL, Parma AE (2010) "Characterization of Shiga toxin-producing *Escherichia coli*  isolated from dairy cows in Argentina." Lett Appl Microbiol 51(4): 377-382.
47. Foster G, Hopkins GF, Gunn GJ, Ternent HE, Thomson-Carter F, Knight HI, Graham DJL, Edge V, Synge BA, (2003) "A comparison of two pre-enrichment media prior to immunomagnetic separation for the isolation of *E. coli* O157 from bovine faeces." J Appl Microbiol 95(1): 155-159.
48. Fox JT, Renter DG, Sanderson MW, Nutsch AL, Shi X, Nagaraja TG (2008) "Associations between the presence and magnitude of *Escherichia coli*  O157 in feces at harvest and contamination of preintervention beef carcasses." J Food Prot 71(9): 1761-1767.
49. Fox JT, Shi X, Nagaraja TG (2008) "*Escherichia coli* O157 in the rectoanal mucosal region of cattle." Foodborne Pathog Dis 5(1): 69-77.
50. Fremaux B, Raynaud S, Beutin L, Rozand CV (2006) "Dissemination and persistence of Shiga toxin-producing *Escherichia coli*  (STEC) strains on French dairy farms." Vet Microbiol 117(2-4): 180-191.
51. Fukushima H, Seki R (2004) "High numbers of Shiga toxin-producing *Escherichia coli*  found in bovine faeces collected at slaughter in Japan." FEMS Microbiol Lett 238(1): 189-197.
52. Garber L, Wells S,Schroeder-tucker L, Ferris K (1999) "Factors associated with fecal shedding of verotoxin-producing *Escherichia coli*  O157 on dairy farms." J Food Prot 62(4): 307-312.
53. Greenquist MA, Drouillard JS,Sargeant JM, Depenbusch BE, Shi X, Lechtenberg KF, Nagaraja TG (2005) "Comparison of rectoanal mucosal swab cultures and fecal cultures for determining prevalence of *Escherichia coli*  O157:H7 in feedlot cattle." Appl Environ Microbiol 71(10): 6431-6433.
54. Gunn GJ, McKendrick IJ,Ternent HE,Thomson-Carter F, Foster G, Synge BA (2007) "An investigation of factors associated with the prevalence of verocytotoxin producing *Escherichia coli*  O157 shedding in Scottish beef cattle." Vet J 174(3): 554-564.
55. Hancock DD, Besser TE, Kinsel ML, Tarr PI, Rice DH, Paros MG (1994) "The prevalence of *Escherichia coli*  O157.H7 in dairy and beef cattle in Washington State." Epidemiol Infect 113(2): 199-207.
56. Hancock DD, Besser TE, Rice DH, Ebel ED, Herriott DE, Carpenter LV (1998) "Multiple sources of *Escherichia coli*  O157 in feedlots and dairy farms in the northwestern USA." Prev Vet Med 35(1): 11-19.
57. Hancock DD, Besser TE, Rice DH, Herriott DE, Tarr PI (1997) "A longitudinal study of *Escherichia coli* O157 in fourteen cattle herds." Epidemiol Infect 118(2): 193-195.
58. Hancock DD, Rice DH, Herriott DE, Besser TE, Ebel E, Carpenter LV (1997) "Effects of farm manure-handling practices on *Escherichia coli* O157 prevalence in cattle." J Food Prot 60(4): 363-366.
59. Heuvelink AE, Van den Biggelaar FLAM, Boer ED, Herbes RG, Melchers WJG, Huis JHJ, Monnens LAH (1998) Isolation and Characterization of Verocytotoxin-Producing *Escherichia coli* O157 Strains from Dutch Cattle and Sheep. J. Clin. Microbiol. 36(4):878.
60. Heuvelink AE, Van den biggelaar FLAM, Zwartkruis-nahuis JTM, Herbes RG, Huyben R, Nagelkerke N, Melchers WJG, Monnens LAH, Boer ED (1998) "Occurrence of verocytotoxin-producing *Escherichia coli* O157 on Dutch dairy farms." J Clin Microbiol 36(12): 3480-3487.
61. Hyatt DR, Galland JC, Gillespie JR (2001) "Usefulness of a commercially available enzyme immunoassay for Shiga-like toxins I and II as a presumptive test for the detection of *Escherichia coli* O157:H7 in cattle feces." J Vet Diagn Invest 13(1): 71-73.
62. Irino K, Katoa MAMF, Vaza TMI, Ramosa II, Souzab MAC, Cruza AS, Gomesc TAT, Vieirac MAM, Guth BEC (2005) "Serotypes and virulence markers of Shiga toxin-producing *Escherichia coli*  (STEC) isolated from dairy cattle in Sao Paulo State, Brazil." Vet Microbiol 105(1): 29-36.
63. Islam MA, Mondol AS, Boer ED, Beumer RR, Zwietering MH, Talukder KH, Heuvelink AE (2008) "Prevalence and genetic characterization of shiga toxin-producing *Escherichia coli*  isolates from slaughtered animals in Bangladesh." Appl Environ Microbiol 74(17): 5414-5421.
64. Jacob ME, Almes KM, Shi X, Sargeant JM, Nagaraja TG (2011) "*Escherichia coli*  O157:H7 genetic diversity in bovine fecal samples." J Food Prot 74(7): 1186-1188.
65. Jacob ME, Renter DG, Nagaraja TG (2010) "Animal- and truckload-level associations between *Escherichia coli* O157:H7 in feces and on hides at harvest and contamination of preevisceration beef carcasses." J Food Prot 73(6): 1030-1037.
66. Jo MY, Kimb JH, Lima JH, Kanga MH, Koha HB, Parkb YH, Yoonc DY, Chaed JS, Eod SK, Lee JH (2004) "Prevalence and characteristics of *Escherichia coli*  O157 from major food animals in Korea." Int J Food Microbiol 95(1): 41-49.
67. Johnsen G, Wasteson Y, Heir E, Berget OI, Herikstad H (2001) "*Escherichia coli*  O157:H7 in faeces from cattle, sheep and pigs in the southwest part of Norway during 1998 and 1999." Int J Food Microbiol 65(3): 193-200.
68. Kang SJ, Ryua SJ, Chaea JS, Eoa SK, Woo GJ, Lee JH (2004) "Occurrence and characteristics of enterohemorrhagic *Escherichia coli*  O157 in calves associated with diarrhoea." Vet Microbiol 98(3-4): 323-328.
69. Keen JE, Durso LM, Meehan TP (2007) "Isolation of Salmonella enterica and Shiga-toxigenic *Escherichia coli* O157 from feces of animals in public contact areas of United States zoological parks." Appl Environ Microbiol 73(1): 362-365.
70. Keen JE, Wittum TE, Dunn JR, Bono JL, Durso LM (2006) "Shiga-toxigenic *Escherichia coli*  O157 in agricultural fair livestock, United States." Emerg Infect Dis 12(5): 780-786.
71. 160. Kerr P, Finlay D, Thomson-Carter F, Ball HJ (2001) "A comparison of a monoclonal antibody-based sandwich ELISA and immunomagnetic bead selective enrichment for the detection of *Escherichia coli*  O157 from bovine faeces." J Appl Microbiol 91(5): 933-936.
72. Kijima-Tanaka M, Ishihara K, Kojima A, Morioka A, Nagata R, Kawanishi M, Nakazawa M,Tamura Y, Takahashi T (2005) "A national surveillance of Shiga toxin-producing *Escherichia coli*  in food-producing animals in Japan." J Vet Med B Infect Dis Vet Public Health 52(5): 230-237.
73. Kobayashi H, Miura A, Hayashi H, Ogawa T, Endo T, Hata E, Eguchi M, Yamamoto K (2003) "Prevalence and characteristics of eae-positive *Escherichia coli*  from healthy cattle in Japan." Appl Environ Microbiol 69(9): 5690-5692.
74. Kuhnert P, Dubosson CR, Roesch M, Homfeld E, Doherr MG, Blum JW (2005) "Prevalence and risk-factor analysis of Shiga toxigenic *Escherichia coli*  in faecal samples of organically and conventionally farmed dairy cattle." Vet Microbiol 109(1-2): 37-45.
75. Laegreid WW, Elder RO, Keen JE (1999) "Prevalence of *Escherichia coli* O157:H7 in range beef calves at weaning." Epidemiol Infect 123(2): 291-298.
76. Lahti E, KeskimaÈki M, Rantala L, HyvoÈnen P, Siitonen A, Honkanen-Buzalski T (2001) "Occurrence of *Escherichia coli*  O157 in Finnish cattle." Vet Microbiol 79(3): 239-251.
77. Laven RA, Ashmore A, Stewart CS (2003) "*Escherichia coli* in the rumen and colon of slaughter cattle, with particular reference to *E. coli* O157." Vet J 165(1): 78-83.
78. LeJeune JT, Besser TE, Rice DH, Berg JL, Stilborn RP, Hancock DD (2004) "Longitudinal study of fecal shedding of *Escherichia coli*  O157:H7 in feedlot cattle: predominance and persistence of specific clonal types despite massive cattle population turnover." Appl Environ Microbiol 70(1): 377-384.
79. LeJeune JT, Hancock D, Wasteson Y, Skjerve E, Urdahl AM (2006) "Comparison of *E. coli* O157 and Shiga toxin-encoding genes (stx) prevalence between Ohio, USA and Norwegian dairy cattle." Int J Food Microbiol 109(1-2): 19-24.
80. Lejeune JT, Kauffman MD (2005) "Effect of sand and sawdust bedding materials on the fecal prevalence of *Escherichia coli* O157:H7 in dairy cows." Appl Environ Microbiol 71(1): 326-330.
81. Leung PH, Yam WC, Ng WWS, Peiris JSM (2001) "The prevalence and characterization of verotoxin-producing *Escherichia coli* isolated from cattle and pigs in an abattoir in Hong Kong." Epidemiol Infect 126(2): 173-179.
82. Lin YL, Chou C, Pan T (2001) "Screening procedure from cattle feces and the prevalence of *Escherichia coli* O157:H7 in Taiwan dairy cattle." J Microbiol Immunol Infect 34(1): 17-24.
83. Low JC, McKendrick IJ, McKechnie C, Fenlon D, Naylor SW, Currie C, Smith DGE, Allison L, Gally DL (2005) "Rectal carriage of enterohemorrhagic *Escherichia coli*  O157 in slaughtered cattle." Appl Environ Microbiol 71(1): 93-97.
84. Lynch MJ, Fox EM, OConnor L, Jordan K, Murphy M (2012) "Surveillance of verocytotoxigenic *Escherichia coli* in Irish bovine dairy herds." Zoonoses Public Health 59(4): 264-271.
85. Madden RH, Murray KA, Gilmour A (2007) "Carriage of four bacterial pathogens by beef cattle in Northern Ireland at time of slaughter." Lett Appl Microbiol 44(2): 115-119.
86. Manna SK, Brahmane MP, Manna C, Batabyal K, Das R (2006) "Occurrence, virulence characteristics and antimicrobial resistance of *Escherichia coli*  O157 in slaughtered cattle and." Lett Appl Microbiol 43(4): 405-409.
87. Masana MO, Leotta GA, Castillo LLD, Dastek BA, Palladino PM, Galli l, Vilacoba E, Carbonari C, Rodriguez HR, Rivas M (2010) "Prevalence, characterization, and genotypic analysis of *Escherichia coli*  O157:H7/NM from selected beef exporting abattoirs of Argentina." J Food Prot 73(4): 649-656.
88. McDonough PL, Rossiter CA, Rebhun RB, Stehman SM, Lein DH, Shin SJ (2000) "Prevalence of *Escherichia coli*  O157:H7 from cull dairy cows in New York state and comparison of culture methods used during preharvest food safety investigations." J Clin Microbiol 38(1): 318-322.
89. McEvoy JM, Doherty AM, Sheridan JJ, Thomson-Carter FM, Garvey P, McGuire L, Blair IS, McDowell DA (2003) "The prevalence and spread of *Escherichia coli*  O157:H7 at a commercial beef abattoir." J Appl Microbiol 95(2): 256-266.
90. Mechie SC, Chapman PA, Siddons CA (1997) "A fifteen month study of *Escherichia coli* O157:H7 in a dairy herd." Epidemiol Infect 118(1): 17-25.
91. Meichtri L, Miliwebsky E, Gioffre A, Chinen I, Baschkier A, Chillemi G, Guthd BEC, Masanaa MO, Cataldic A, Rodrıgueza HR, Rivas M (2004) "Shiga toxin-producing *Escherichia coli*  in healthy young beef steers from Argentina: prevalence and virulence properties." Int J Food Microbiol 96(2): 189-198.
92. Milnes AS, Stewart I, Clifton-hadley FA, Davies RH, Newell DG, Sayers AR, Cheasty T, Cassar C, Ridley A, Cook AJC, Evans SJ, Teale CJ, Smith RP, Mcnally A, Toszeghy M, Futter R, KAY A, Paiba GA (2008) "Intestinal carriage of verocytotoxigenic *Escherichia coli* O157, Salmonella, thermophilic Campylobacter and Yersinia enterocolitica, in cattle, sheep and pigs at slaughter in Great Britain during 2003." Epidemiol Infect 136(6): 739-751.
93. Minihan D, O’Mahony M, Whyte P, Collins JD (2003) "An investigation on the effect of transport and lairage on the faecal shedding prevalence of *Escherichia coli* O157 in cattle." J Vet Med B Infect Dis Vet Public Health 50(8): 378-382.
94. Miyao Y, Kataokaa T, Nomotoa T, Kaib A, Itohb T, Itoh K (1998) "Prevalence of verotoxin-producing *Escherichia coli*  harbored in the intestine of cattle in Japan." Vet Microbiol 61(1-2): 137-143.
    1. .
95. Montenegro MA, Bülte M, Trumpf T, Aleksic S, Reuter G, Bulling E, Helmuth R (1990) "Detection and characterization of fecal verotoxin-producing *Escherichia coli*  from healthy cattle." J Clin Microbiol 28(6): 1417-1421.
96. Moreira CN, Pereirac MA, Broda CS, Rodriguesd DP, Carvalhalc JB, Aleixo JAG (2003) "Shiga toxin-producing *Escherichia coli*  (STEC) isolated from healthy dairy cattle in southern Brazil." Vet Microbiol 93(3): 179-183.
97. Murinda SE, Nguyen LT, Ivey SJ, Gillespie BE, Almeida RA, Draughon FA, Oliver SP (2002) "Prevalence and molecular characterization of *Escherichia coli*  O157:H7 in bulk tank milk and fecal samples from cull cows: a 12-month survey of dairy farms in east Tennessee." J Food Prot 65(5): 752-759.
98. Murinda SE, Nguyen LT, Nam HM, Almeida RA, Headrick SJ, Oliver SP (2004) "Detection of sorbitol-negative and sorbitol-positive Shiga toxin-producing *Escherichia coli* , Listeria monocytogenes, Campylobacter jejuni, and Salmonella spp. in dairy farm environmental samples." Foodborne Pathog Dis 1(2): 97-104.
99. Narvaez-Bravo CA, Carruyo-Núñez G, MorenoM, Rodas-González A, Hoet AE, Wittum TE (2007) "Isolation of *Escherichia coli*  O157 : H7 from feces in dual purpose cattle at Miranda Municipality, Zulia State, Venezuela." Revista Cientifica-Facultad De Ciencias Veterinarias 17(3): 239-245.
100. Nastasijevic I, Mitrovic R, Buncic S (2009) "The occurrence of *Escherichia coli* O157 in/on faeces, carcasses and fresh meats from cattle." Meat Sci 82(1): 101-105.
101. Nguyen TD, Vo TT, Vu-Khac H (2011) "Virulence factors in *Escherichia coli* isolated from calves with diarrhea in Vietnam." J Vet Sci 12(2): 159-164.
102. Nielsen EM, Tegtmeiera C, Andersen HJ, Grønbæk C, Andersen JS (2002) "Influence of age, sex and herd characteristics on the occurrence of Verocytotoxin-producing *Escherichia coli*  O157 in Danish dairy farms." Vet Microbiol 88(3): 245-257.
103. Ogden ID, MacRae M, Strachan NJC (2004) "Is the prevalence and shedding concentrations of *E. coli* O157 in beef cattle in Scotland seasonal?" FEMS Microbiol Lett 233(2): 297-300.
104. Ojo OE, Ajuwape ATP, Otesile EB, Owoade AA, Oyekunle MA, Adetosoye AI (2010) "Potentially zoonotic shiga toxin-producing *Escherichia coli*  serogroups in the faeces and meat of food-producing animals in Ibadan, Nigeria." Int J Food Microbiol 142(1-2): 214-221.
105. Omisakin F, MacRae M, Ogden ID, Strachan NJC (2003) "Concentration and prevalence of *Escherichia coli* O157 in cattle feces at slaughter." Appl Environ Microbiol 69(5): 2444-2447.
106. Ongor H, Kalin R, Cetinkaya B (2007) "Investigation of *Escherichia coli* O157 and some virulence genes in samples of meat and faeces from clinically healthy cattle in Turkey." Veterinary Record 161(11): 392-394.
107. Oot RA, Raya RR, Callaway TR, Edrington TS, Kutter EM, Brabban AD (2007) "Prevalence of *Escherichia coli*  O157 and O157:H7-infecting bacteriophages in feedlot cattle feces." Lett Appl Microbiol 45(4): 445-453.
108. Osaili TM, Alaboudi AR, Rahahlah M (2013) "Prevalence and antimicrobial susceptibility of *Escherichia coli* O157: H7 on beef cattle slaughtered in Amman abattoir." Meat Sci 93(3): 463-468.
109. Paiba GA, Gibbens JC, Pascoe SJS, Wilesmith JW, Kidd SA, Byrne C, Ryan JBM, Smith RR, Mclaren M, Futter RJ, Kay ACS, Jones YE, Chappell SA, Willshaw GA, Cheasty T (2002) "Faecal carriage of verocytotoxin-producing *Escherichia coli*  O157 in cattle and sheep at slaughter in Great Britain." Vet Rec 150(19): 593-598.
110. Paiba GA, Wilesmith JW, Evans SJ, Pascoe SJS, Smith RP, Kidd SA, Ryan JBM, Mclaren im, Chappell SA, Willshaw GA, Cheasty T, French NP, Jones TWH, Buchanan HF, Challoner DJ, Colloff AD, Cranwell MP, Daniel RG, Davies IH, Duff JP, Hogg RAT, Kirby FD, Millar MF, Monies RJ, Nicholls MJ, Payne JH, (2003) "Prevalence of faecal excretion of verocytotoxigenic *Escherichia coli*  O157 in cattle in England and Wales." Vet Rec 153(12): 347-353.
111. Panutdaporn N, Chongsa-nguan M, Nair GB, Ramamurthy T, Yamasaki S, Chaisri U, Tongtawe P, Eampokalarp B, Tapchaisri P, Sakolvaree Y, Kurazonog H, Thein WB, Hayashi H, Takeda Y, Chaicumpa W (2004) "Genotypes and phenotypes of Shiga toxin producing-*Escherichia coli*  isolated from healthy cattle in Thailand." J Infect 48(2): 149-160.
112. Pearce MC, Chase-Topping ME, McKendrick IJ, Mellor DJ, Locking ME, Allison L, Ternent HE, Matthews L, Knight HI, Smith AW, Synge BA, Reilly W, Low JC, Reid SWJ, Gunn GJ, Woolhouse MEJ (2009) "Temporal and spatial patterns of bovine *Escherichia coli* O157 prevalence and comparison of temporal changes in the patterns of phage types associated with bovine shedding and human *E. coli* O157 cases in Scotland between 1998-2000 and 2002-2004." BMC Microbiol 9: 276.
113. Pradel N, Livrelli V, Champs CD, Palcoux JB, Reynaud A, Scheutz F, Sirot J, Joly B, Forestier C (2000) "Prevalence and characterization of Shiga toxin-producing *Escherichia coli*  isolated from cattle, food, and children during a one-year prospective study in France." J Clin Microbiol 38(3): 1023-1031.
114. Ransom JR, Belk KE, Bacon RT, Sofos JN, Scanga JA, Smith GC (2002) "Comparison of sampling methods for microbiological testing of beef animal rectal/colonal feces, hides, and carcasses." J Food Prot 65(4): 621-626.
115. Renter DG, Sargeant JM, Hungerford LL, (2004) "Distribution of *Escherichia coli* O157:H7 within and among cattle operations in pasture-based agricultural areas." Am J Vet Res 65(10): 1367-1376.
116. Richards MS, Corkish JD, Sayers AR, McLaren IM, Evans SJ, Wray C (1998) "Studies of the presence of verocytotoxic *Escherichia coli*  O157 in bovine faeces submitted for diagnostic purposes in England and Wales and on beef carcases in abattoirs in the United Kingdom." Epidemiol Infect 120(2): 187-192.
117. Rivera FP, Sotelo E, Morales I, Menacho F, Medina AM, Evaristo R, Valencia R, Carbajal L, Ruiz J, Ochoa TJ (2012) "Short communication: Detection of Shiga toxin-producing *Escherichia coli*  (STEC) in healthy cattle and pigs in Lima, Peru." J Dairy Sci 95(3): 1166-1169.
118. Sanchez S, Martínez R, García A, Blanco J, Echeita A, Hermoso J, Mendoza D, Rey J, Alonso JM (2010) "Shiga toxin-producing *Escherichia coli*  O157:H7 from extensive cattle of the fighting bulls breed." Res Vet Sci 88(2): 208-210.
119. Sargeant JM, Sandersonb MW, Smithc RA, Griffin DD (2003) "*Escherichia coli* O157 in feedlot cattle feces and water in four major feeder-cattle states in the USA." Prev Vet Med 61(2): 127-135.
120. Sasaki Y, Tsujiyama Y, Kusukawa M, Murakami M, Katayama S, Yamada Y (2011) Prevalence and characterization of Shiga toxin-producing *Escherichia coli* O157 and O26 in beef farms. Veterinary Microbiology 150, 140–145.
121. Schurman RD, Hariharan H, Heaney SB, Rahn K (2000) "Prevalence and characteristics of shiga toxin-producing *Escherichia coli*  in beef cattle slaughtered on Prince Edward Island." J Food Prot 63(11): 1583-1586.
122. Shinagawa K, Kanehira M, Omoe K, Matsuda I, Hu DL, Widiasih DA, Sugiib S (2010) Frequency of Shiga toxin-producing *Escherichia coli*  in cattle at a breeding farm and at a slaughterhouse in Japan. Vet. Microbiol. 76, 305–309.
123. Sisti M, Benedetti C, Lonzi A, Schiavano GF, Pianetti A, Romanini I, Bruscolini F (2004) "Isolation of *Escherichia coli*  O157 from human and bovine faeces in the Urbino area, Italy." Int J Hyg Environ Health 207(6): 577-583.
124. Smith D, Blackford M, Younts S, Moxley R, Gray J, Hungerford L, Milton T, Klopfenstein T (2001) "Ecological relationships between the prevalence of cattle shedding *Escherichia coli*  O157:H7 and characteristics of the cattle or conditions of the feedlot pen." J Food Prot 64(12): 1899-1903.
125. Tahamtan Y, Hayati M, Namavari MM (2010) "Prevalence and distribution of the stx, stx genes in Shiga toxin producing *E. coli* (STEC) isolates from cattle." Iranian journal of microbiology 2(1), 9-14.
126. Tanaro JD, Leotta GA, Lound LH, Galli L, Piaggio MC, Carbonari CC, Araujo S, Rivas M, (2010) "*Escherichia coli*  O157 in bovine feces and surface water streams in a beef cattle farm of Argentina." Foodborne Pathog Dis 7(4): 475-477.
127. Thomas KM, McCann MS, Collery MM, Logan A, Whyte P, McDowell DA, Duffy G (2012) "Tracking verocytotoxigenic *Escherichia coli* O157, O26, O111, O103 and O145 in Irish cattle." Int J Food Microbiol 153(3): 288-296.
128. Tutenel AV, Pierard D, Uradzinski J, Jozwik E, Pastuszczak M, Hende JV, Uyttendaele M, Debevere J, Cheasty T, Hoof JV, Zutter LD (2002) Isolation and characterization of enterohaemorrhagic *Escherichia coli*  O157:H7 from cattle in Belgium and Poland. Epidemiol Infect. 129, 41-47.
129. Van Donkersgoed J, Graham T, Gannon V (1999) "The prevalence of verotoxins, *Escherichia coli* O157:H7, and Salmonella in the feces and rumen of cattle at processing." Can Vet J 40(5): 332-338.
130. Vernozy-Rozand C, FengP, Montet MP, Ray-Gueniot S, Villard L, Bavai C, Meyrand A, Mazuy C, Atrache V (2000) "Detection of *Escherichia coli*  O157:H7 in heifers' faecal samples using an automated immunoconcentration system." Lett Appl Microbiol 30(3): 217-222.
131. Vidovic S, Korber DR (2006) "Prevalence of *Escherichia coli* O157 in Saskatchewan cattle: characterization of isolates by using random amplified polymorphic DNA PCR, antibiotic resistance profiles, and pathogenicity determinants." Appl Environ Microbiol 72(6): 4347-4355.
132. Vold L, klungseth B, Johansen, Kruse H, Skjerve E, Wasteson Y (1998) Occurrence of shigatoxinogenic *Escherichia coli*  O157 in Norwegian cattle herds. Epidemiol. Infect. 120, 21-28.
133. Vuddhakul V, Patararungrong N, Pungrasamee P, Jitsurong S, Morigaki T, Asai N, Nishibuchi M (2000) "Isolation and characterization of *Escherichia coli*  O157 from retail beef and bovine feces in Thailand." FEMS Microbiol Lett 182(2): 343-347.
134. Walker C, Shi X, Sanderson M, Sargeant J, Nagaraja TG (2010) "Prevalence of *Escherichia coli*  O157:H7 in gut contents of beef cattle at slaughter." Foodborne Pathog Dis 7(3): 249-255.
135. Wani SA, Bhat MA, Samanta, Nishikawa Y, Buchh AS (2003) "Isolation and characterization of Shiga toxin-producing *Escherichia coli* (STEC) and enteropathogenic *Escherichia coli*  (EPEC) from calves and lambs with diarrhoea in India." Lett Appl Microbiol 37(2): 121-126.
136. Wells JG, Shipman LD, Greene KD, Sowers EG, Green JH, NCameron D, Downes FP, Martin ML, Griffin PM, Ostroff SM (1991) "isolation of Escherichia-coli serotype o157 h7 and other shiga-like-toxin-producing escherichia-coli from dairy cattle." J Clin Microbiol 29(5): 985-989.
137. Widiasih DA, Ido N, Omoe K, Sugii S, Shinagawa K (2004) "Duration and magnitude of faecal shedding of Shiga toxin-producing *Escherichia coli*  from naturally infected cattle." Epidemiol Infect 132(1): 67-75.
138. Woerner DR, Ransom JR, Sofos JN, Dewell GA, Smith GC, Salman MD, Belk KE (2006) "Determining the prevalence of *Escherichia coli*  O157 in cattle and beef from the feedlot to the cooler." J Food Prot 69(12): 2824-2827.
139. Yilmaz A, Gun H, Yilmaz H (2002) "Frequency of *Escherichia coli* O157:H7 in Turkish cattle." J Food Prot 65(10): 1637-1640.
140. Zhou Z, Nishikawa Y, Zhu P, Hong S, Hase A, Cheasty T, Smith HR, Zheng M, Haruki K (2002) "Isolation and characterization of Shiga toxin-producing *Escherichia coli*  O157:H7 from beef, pork and cattle fecal samples in Changchun, China." J Vet Med Sci 64(11): 1041-1044.
