# Supplementary material for: Regional Variation in the Prevalence of E. coli O157 in Cattle: A Meta-Analysis and Meta-Regression
Source: PLoS One. 2014 Apr 1;9(4):e93299. doi: 10.1371/journal.pone.0093299 (PMC3972218; doi:10.1371/journal.pone.0093299)
Supplement: File S4 — List of excluded full text paper with proper justification. (DOC) [file pone.0093299.s005.doc]

**Articles excluded from this study (with justification)**

**N=107**

**Case report (n=1)**

1. Jackson SG, Goodbrand RB, Johnson RP, Odorico VG, Alves D, Rahn K, Wilson JB, Welch MK, Khakhria R (1998) "*Escherichia coli* O157:H7 diarrhoea associated with well water and infected cattle on an Ontario farm." Epidemiol Infect 120(1): 17-20.

**Experimental trial (n=15)**

1. Arthur TM, Bosilevac JM, Kalchayanand N, Wells JE, Shackelford SD, Wheeler TL, Koohmaraie M (2010). "Evaluation of a direct-fed microbial product effect on the prevalence and load of *Escherichia coli* O157:H7 in feedlot cattle." J Food Prot 73(2): 366-371.
2. Berry ED, Wells JE, Arthur TM, Woodbury BL, Nienaber JA, Brown-brandl TM, Eigenberg RA (2010) "Soil versus Pond Ash Surfacing of Feedlot Pens: Occurrence of *Escherichia coli* O157:H7 in Cattle and Persistence in Manure." J Food Prot 73(7): 1269-1277.
3. Braden KW, Blanton jr. JR, Allen VG, Pond KR, Miller MF, (2004) "Ascophyllum nodosum supplementation: a preharvest intervention for reducing *Escherichia coli* O157:H7 and Salmonella spp. in feedlot steers." J Food Prot 67(9): 1824-1828.
4. Brashears MM, Galyean ML, Loneragan GH, Mann JE, Killinger-mann K (2003) "Prevalence of *Escherichia coli* O157:H7 and performance by beef feedlot cattle given Lactobacillus direct-fed microbials." J Food Prot 66(5): 748-754.
5. Brown-Brandl TM, Berry ED, Wells JE, Arthur TM, Nienaber JA (2009) "Impacts of individual animal response to heat and handling stresses on *Escherichia coli* and E. coli O157:H7 fecal shedding by feedlot cattle." Foodborne Pathog Dis 6(7): 855-864.
6. Cray WC Jr, Casey TA, Bosworth BT, Rasmussen MA (1998). "Effect of dietary stress on fecal shedding of *Escherichia coli* O157:H7 in calves." Appl Environ Microbiol 64(5): 1975-1979.
7. Depenbusch BE, Nagaraja TG, Sargeant, JM, Drouillard JS, Loe ER, Corrigan ME (2008) "Influence of processed grains on fecal pH, starch concentration, and shedding of *Escherichia coli* O157 in feedlot cattle." J Anim Sci 86(3): 632-639.
8. Edrington TS, Carter BH, Farrow RL, Islas A, Hagevoort GR, Friend TH, Callaway TR, Anderson RC, Nisbet DJ (2011) "Influence of weaning on fecal shedding of pathogenic bacteria in dairy calves." Foodborne Pathog Dis 8(3): 395-401.

1. Edrington TS, Farrow R, Loneragan GH, Ives SE, Engler MJ, Wagner JJ, Corbin MJ, Platter WJ, Yates D, Hutcheson JP, Zinn RA, Callaway TR, Anderson RC, Nisbet DJ (2009) "Influence of beta-agonists (ractopamine HCl and zilpaterol HCl) on fecal shedding of *Escherichia coli* O157:H7 in feedlot cattle." J Food Prot 72(12): 2587-2591.
2. Edrington TS, Farrow RL, Mackinnon KM, Callaway TR, Anderson RC, Nisbet DJ (2012) "Influence of vitamin D on fecal shedding of *Escherichia coli* O157:H7 in naturally colonized cattle." J Food Prot 75(2): 314-319.
3. Edrington TS, Looper ML, Duke SE, Callaway TR, Genovese KJ, Anderson RC, Nisbet DJ (2006) "Effect of ionophore supplementation on the incidence of *Escherichia coli* O157:H7 and Salmonella and antimicrobial susceptibility of fecal coliforms in Stocker cattle." Foodborne Pathog Dis 3(3): 284-291.
4. Edrington TS, Schultz CL, Genovese KJ, Callaway TR, Looper ML, Bischoff KM, Mcreynolds JL, Anderson RC, Nisbet DJ (2004) "Examination of heat stress and stage of lactation (early versus late) on fecal shedding of E. coli O157:H7 and Salmonella in dairy cattle." Foodborne Pathog Dis 1(2): 114-119.
5. Robinson SE, Wright EJ, Hart CA, Bennett M, French NP (2004) "Intermittent and persistent shedding of *Escherichia coli* O157 in cohorts of naturally infected calves." J Appl Microbiol 97(5): 1045-1053.
6. Stephens TP, Loneragan GH, Chichester LM, Brashears MM (2007) "Prevalence and enumeration of *Escherichia coli* O157 in steers receiving various strains of Lactobacillus-based direct-fed microbials." J Food Prot 70(5): 1252-1255.
7. Wells JE, Shackelford SD, Berry ED, Kalchayanand N, Guerini MN, Varel VH, Arthur TM, Bosilevac JM, Freetly HC, Wheeler TL, Ferrell CL, Koohmaraie M, (2009) "Prevalence and level of *Escherichia coli* O157:H7 in feces and on hides of feedlot steers fed diets with or without wet distillers grains with solubles." J Food Prot 72(8): 1624-1633.

**Farm level prevalence (n=3)**

1. Chase-Topping ME, McKendrick IJ, Pearce MC, MacDonald P, Matthews L, Halliday J, Allison L, Fenlon D, Christopher Low J, Gunn G, Woolhouse MEJ (2007) "Risk factors for the presence of high-level shedders of *Escherichia coli* O157 on Scottish farms." J Clin Microbiol 45(5): 1594-1603.
2. Cobbaut K, Berkvens D, Houf K, Deken R.D, Zutter LD (2009) "*Escherichia coli* O157 prevalence in different cattle farm types and identification of potential risk factors." J Food Prot 72(9): 1848-1853.
3. Cobbaut K,Houf K, Buvens G,Habib I, Zutter LD (2009) "Occurrence of non-sorbitol fermenting, verocytotoxin-lacking *Escherichia coli* O157 on cattle farms." Vet Microbiol 138(1-2): 174-178.

**Individual prevalence data is not available (n=3)**

1. Berg J, Mcallister T, Bach S, Stilborn R, Hancock D, Lejeune J (2004) "*Escherichia coli* O157:H7 excretion by commercial feedlot cattle fed either barley- or corn-based finishing diets." J Food Prot 67(4): 666-671.
2. Renter DG, Smith DR, King R, Stilborn R, Berg J, Berezowski J, McFall M (2008) "Detection and determinants of *Escherichia coli* O157:H7 in Alberta feedlot pens immediately prior to slaughter." Can J Vet Res 72(3): 217-227.
3. Riley DG, Gray JT, Loneragan GH, Barling KS, Chase CC jr. (2003). "*Escherichia coli* O157:H7 prevalence in fecal samples of cattle from a southeastern beef cow-calf herd." J Food Prot 66(10): 1778-1782.

**Pen level prevalence (n=3)**

1. Dewell GA, Ransom JR, Dewell RD, Mccurdy K, Gardner IA, Hill AE, Sofos JE, Belk KE, Smith GC, Salman MD (2005). "Prevalence of and risk factors for *Escherichia coli* O157 in market-ready beef cattle from 12 U.S. feedlots." Foodborne Pathog Dis 2(1): 70-76.
2. Van Donkersgoed J, Berg J, Potter A, Hancock D, Besser T, Rice D, LeJeune J, Klashinsky S (2001) "Environmental sources and transmission of *Escherichia coli* O157 in feedlot cattle." Can Vet J 42(9): 714-720.
3. Van Donkersgoed J, Bohaychuk V, Besser T, Song X, Wagner B, Hancock D, Renter D, Dargatz D (2009) "Occurrence of foodborne bacteria in Alberta feedlots." Can Vet J 50(2): 166-172.

**Pooled samples (n=9)**

1. Bolton DJ, O’Neill CJ. Fanning S (2012) "A preliminary study of Salmonella, verocytotoxigenic Escherichia coli/*Escherichia coli* O157 and Campylobacter on four mixed farms." Zoonoses Public Health 59(3): 217-228.
2. lin YL, Chou CC, Pan TM (2001) "Screening procedure from cattle feces and the prevalence of *Escherichia coli* O157 in Taiwan's dairy cattle." Biomedical and Environmental Sciences 14(1-2): 153-154.
3. Ellis-Iversen J, Smith RP, Snowa LC, Watson E, Millar MF, Pritchard GC, Sayers AR, Cook AJC, Evans SJ, Paiba GA (2007) "Identification of management risk factors for VTEC O157 in young-stock in England and Wales." Prev Vet Med 82(1-2): 29-41.
4. Eriksson E, Aspan A, Gunnarsson A, Gsholm IV (2005) "Prevalence of verotoxin-producing *Escherichia coli* (VTEC) 0157 in Swedish dairy herds." Epidemiol Infect 133(2): 349-358.
5. Galland JC, Hyatt DR, Crupper SS, Acheson DW (2001) "Prevalence, antibiotic susceptibility, and diversity of *Escherichia coli* O157:H7 isolates from a longitudinal study of beef cattle feedlots." Appl Environ Microbiol 67(4): 1619-1627.
6. Renter DG, Checkley SL, Campbell J, King R (2004) "Shiga toxin-producing *Escherichia coli* in the feces of Alberta feedlot cattle." Can J Vet Res 68(2): 150-153.
7. Sami M, Firouzi R, Shekarforoush SS (2007) "Prevalence of *Escherichia coli* O157: H7 on dairy farms in Shiraz, Iran by immunomagnetic separation and multiplex PCR." Iranian Journal of Veterinary Research 8(4): 319-324.
8. Schouten JM, Bouwknegt M, Giessen AWV, Frankena K, De Jong MCM, Graat EAM, (2004) Prevalence estimation and risk factors for *Escherichia coli* O157 on Dutch dairy farms Preventive Veterinary Medicine 64, 49–61.
9. Schouten JM, Giessen AWV, Frankena K, De Jong MCM, Graat EAM (2005) *Escherichia coli* O157 prevalence in Dutch poultry, pig finishing and veal herds and risk factors in Dutch veal herds. Preventive Veterinary Medicine 70, 1–15.

**Prevalence data not available (n=28)**

1. Aslam M, Stanford K, McAllisteret TA (2010) "Characterization of antimicrobial resistance and seasonal prevalence of *Escherichia coli* O157:H7 recovered from commercial feedlots in Alberta, Canada." Lett Appl Microbiol 50(3): 320-326.
2. Bach SJ, Mcallister TA, Mears GJ, Schwartzkopf-genswein KS, (2004) "Long-haul transport and lack of preconditioning increases fecal shedding of *Escherichia coli* and *Escherichia coli* O157:H7 by calves." J Food Prot 67(4): 672-678.
3. Bardiau M, Muylaert A, Duprez JN, Labrozzo S, Mainil JG (2010) "Prevalence, molecular typing, and antibiotic sensitivity of enteropathogenic, enterohaemorrhagic, and verotoxigenic *Escherichia coli* isolated from veal calves." Tijdschr Diergeneeskd 135(14-15): 554-558.
4. Blanco M, Blanco JE, Mora A, Dahbi G, Alonso MP, Gonza´lez EA, Berna´rdez MI, Blanco J (2004) "Serotypes, virulence genes, and intimin types of Shiga toxin (verotoxin)-producing *Escherichia coli* isolates from cattle in Spain and identification of a new intimin variant gene (eae-xi)." J Clin Microbiol 42(2): 645-651.
5. Chapman P A, Malo AT, Siddons CA, Harkin M (1997) "Use of commercial enzyme immunoassays and immunomagnetic separation systems for detecting *Escherichia coli* O157 in bovine fecal samples." Appl Environ Microbiol 63(7): 2549-2553.
6. Cobbold RN, Rice DH, Szymanski M, Call DR, Hancock DD (2004) "Comparison of shiga-toxigenic *Escherichia coli* prevalences among dairy, feedlot, and cow-calf herds in Washington State." Appl Environ Microbiol 70(7): 4375-4378.
7. D'Astek BA,Castillo LLD, Miliwebsky E, Carbonari C, Palladino PM, Deza N, Chinen I, Manfredi E, Leotta GA, Masana MO, Rivas M (2012) "Subtyping of *Escherichia coli* O157:H7 strains isolated from human infections and healthy cattle in Argentina." Foodborne Pathog Dis 9(5): 457-464.
8. Dutta S, Deb A, Chattapadhyay UK, Tsukamoto T, (2000) "Isolation of Shiga toxin-producing *Escherichia coli* including O157:H7 strains from dairy cattle and beef samples marketed in Calcutta, India." J Med Microbiol 49(8): 765-767.
9. Fegan N, Higgs G, Vanderlinde P, Desmarchelier P (2005) "An investigation of *Escherichia coli* O157 contamination of cattle during slaughter at an abattoir." J Food Prot 68(3): 451-457.
10. Feng PCH, Keys C, Lacher D, Monday SR, Shelton D, Rozand C, Rivas M, Whittam T (2010) "Prevalence, characterization and clonal analysis of *Escherichia coli* O157: non-H7 serotypes that carry eae alleles." FEMS Microbiol Lett 308(1): 62-67.
11. Fox JT, Depenbusch BE, Drouillard JS, Nagaraja TG (2007) "Dry-rolled or steam-flaked grain-based diets and fecal shedding of *Escherichia coli* O157 in feedlot cattle." J Anim Sci 85(5): 1207-1212.
12. Geue L, Segura-alvarez M, Conraths FJ, Kuczius T, Bockemu J, Karch H, Gallien P (2002) "A long-term study on the prevalence of shiga toxin-producing *Escherichia coli* (STEC) on four German cattle farms." Epidemiol Infect 129(1): 173-185.
13. Guth BEC, Chinen I, Miliwebsky E, Cerqueira AMF, Chillemi G, Andrade JRC, Baschkier A, Rivas M (2003) "Serotypes and Shiga toxin genotypes among *Escherichia coli* isolated from animals and food in Argentina and Brazil." Vet Microbiol 92(4): 335-349.
14. Halliday JEB, Chase-Topping ME, Pearce MC, McKendrick MJ, Allison L, Fenlon D, Low C, Mellor DJ, GunnGJ,Woolhouse MEJ (2006) "Herd-level risk factors associated with the presence of Phage type 21/28 E. coli O157 on Scottish cattle farms." BMC Microbiol 6: 99.
15. Kim JY, Kim S, Kwon N, Bae W, Lim J, Koo H, Kim J, Noh K, Jung W, Park K, Park Y, (2005) "Isolation and identification of *Escherichia coli* O157:H7 using different detection methods and molecular determination by multiplex PCR and RAPD." J Vet Sci 6(1): 7-19.
16. Kistemann T, Zimmer S, Vagsholm I, Andersson Y (2004) "GIS-supported investigation of human EHEC and cattle VTEC O157 infections in Sweden: geographical distribution, spatial variation and possible risk factors." Epidemiol Infect 132(3): 495-505.
17. Niu YD, McAllister TA, Xu Y, Johnson RP, Stephens TP, Stanford K (2009) "Prevalence and impact of bacteriophages on the presence of *Escherichia coli* O157:H7 in feedlot cattle and their environment." Appl Environ Microbiol 75(5): 1271-1278.
18. Oberst RD, Hays MP, Bohra LK, Phebus RK, Sargeant JM (2003) "Detection of *Escherichia coli* O157:H7 in cattle feces using a polymerase chain reaction-based fluorogenic 5' nuclease (TaqMan) detection assay after secondary enrichment." J Vet Diagn Invest 15(6): 543-552.
19. Pearce MC, Fenlon D, Low JC, Smith AW, Knight HI, Evans J, Foster G, Synge BA, Gunn GJ (2004) "Distribution of *Escherichia coli* O157 in bovine fecal pats and its impact on estimates of the prevalence of fecal shedding." Appl Environ Microbiol 70(10): 5737-5743.
20. Reinstein S, Fox JT, Shi X, Alam MJ, Renter DG, Nagaraja TG (2009) "Prevalence of *Escherichia coli* O157:H7 in organically and naturally raised beef cattle." Appl Environ Microbiol 75(16): 5421-5423.
21. Renter DG, Morris JG jr., Sargeant JM, Hungerford LL, Berezowsk J, Ngo T, Williams K, Acheson DWK (2005) "Prevalence, risk factors, O serogroups, and virulence profiles of Shiga toxin-producing bacteria from cattle production environments." J Food Prot 68(8): 1556-1565.
22. Robinson SE, Brown PE, Wright EJ, Hart CA, French NP (2009) "Quantifying within- and between-animal variation and uncertainty associated with counts of *Escherichia coli* O157 occurring in naturally infected cattle faeces." J R Soc Interface 6(31): 169-177.
23. Roopnarine RR, Ammons D, Rampersad J, Adesiyun AA (2007) "Occurrence and characterization of verocytotoxigenic *Escherichia coli* (VTEC) strains from dairy farms in Trinidad." Zoonoses Public Health 54(2): 78-85.
24. Vosough Ahmadi B, Klaas F, Joanne T, Annet GJV, Henk H, Ruud BMH, (2007) "Effectiveness of simulated interventions in reducing the estimated prevalence of E. coli O157:H7 in lactating cows in dairy herds." Vet Res 38(5): 755-771.
25. Vu-Khac H, Cornick NA (2008) "Prevalence and genetic profiles of Shiga toxin-producing *Escherichia coli* strains isolated from buffaloes, cattle, and goats in central Vietnam." Vet Microbiol 126(4): 356-363.
26. Wallace JS, Jones K (1996) "The use of selective and differential agars in the isolation of *Escherichia coli* O157 from dairy herds." J Appl Bacteriol 81(6): 663-668.
27. Yilmaz A, Gun H, Ugur M, Turan N, Yilmaz H (2006) "Detection and frequency of VT1, VT2 and eaeA genes in *Escherichia coli* O157 and O157 : H17 strains isolated from cattle, cattle carcasses." Int J Food Microbiol 106(2): 213-217.
28. Fitzgerald AC, Edrington TS, Looper ML, Callaway TR, Genovese KJ, Bischoff KM, McReynolds JL, Thomas JD, Anderson RC, Nisbet DJ (2002) Antimicrobial susceptibility and factors affecting the shedding of E. coli O157:H7 and Salmonella in dairy cattle. Letters in Applied Microbiology 37, 392–398.

**No positive case reported (n=4)**

1. Al-Charrakh A, Al-Muhana A ( 2010) Prevalence of verotoxin-producing *Escherichia coli* (VTEC) in a survey of dairy cattle in Najaf, Iraq. Iranian Journal of Microbiology, Volume 2 Number 3 (September 2010) 130-136.
2. Kaddu-Mulindw DH, Aisu T, Gleier K, Zimmermann S, Beutin L (2001) "Occurrence of Shiga toxin-producing *Escherichia coli* in fecal samples from children with diarrhea and from healthy zebu cattle in Uganda." Int J Food Microbiol 66(1-2): 95-101.
3. Karama M, Johnson RP, Holtslander R, McEwen SA, Gyles CL (2008) "Prevalence and characterization of verotoxin-producing *Escherichia coli* (VTEC) in cattle from an Ontario abattoir." Can J Vet Res 72(4): 297-302.
4. Zschock M, Hamann HP, Kloppert B, Wolter W (2000) "Shiga-toxin-producing *Escherichia coli* in faeces of healthy dairy cows, sheep and goats: prevalence and virulence properties." Lett Appl Microbiol 31(3): 203-208.

**Sample from previously known (E. coli o157) sources (n=5)**

1. Ellis-Iversen J, Cook AJC, Smith RP, Pritchard GC, Nielen M (2009) "Temporal patterns and risk factors for *Escherichia coli* O157 and Campylobacter spp, in young cattle." J Food Prot 72(3): 490-496.
2. Schouten JM, Graat EAM, Frankena K, Giessen AWV, Zwaluw WKV, De Jong MCM (2005). "A longitudinal study of *Escherichia coli* O157 in cattle of a Dutch dairy farm and in the farm environment." Vet Microbiol 107(3-4): 193-204.
3. Smith RP, Paiba GA, Ellis-Iversen J (2010) "Longitudinal study to investigate VTEC O157 shedding patterns in young cattle." Res Vet Sci 88(3): 411-414.
4. Wasteson Y, Johannessen GS, Bruheim T, Urdahl AM, O’Sullivan K, Rørvik LM (2005) "Fluctuations in the occurrence of *Escherichia coli* O157:H7 on a Norwegian farm*." Lett Appl Microbiol 40(5): 373-377.
5. Zhao T, Doyle MP, Shere J, Garber L (1995) "Prevalence of enterohemorrhagic *Escherichia coli* O157:H7 in a survey of dairy herds." Appl Environ Microbiol 61(4): 1290-1293.

**Repeated sample from same animal/animal group (n=13)**

1. Chapman PA, Wright DJ, Siddons CA (1994). "A comparison of immunomagnetic separation and direct culture for the isolation of verocytotoxin-producing *Escherichia coli* O157 from bovine faeces." J Med Microbiol 40(6): 424-427.
2. Edrington TS, Farrow RL, Sperandio V, Hughes DT, Lawrence TE, Callaway TR, Anderson RC, Nisbet DJ (2009) "Acyl-homoserine-lactone autoinducer in the gastrointestinal [corrected] tract of feedlot cattle and correlation to season, E. coli O157:H7 prevalence, and diet." Curr Microbiol 58(3): 227-232.
3. Edrington TS, Hume ME, Looper ML, Schultz CL, Fitzgerald AC, Callaway TR, Genovese KJ, Bischoff KM, McReynolds JL, Anderson RC, Nisbet DJ (2004) "Variation in the faecal shedding of Salmonella and E. coli O157:H7 in lactating dairy cattle and examination of Salmonella genotypes using pulsed-field gel electrophoresis." Lett Appl Microbiol 38(5): 366-372.
4. Gannon VPJ, Graham TA, King R, Michel P, Read S, Ziebell K, Johnson RP (2002) "*Escherichia coli* O157:H7 infection in cows and calves in a beef cattle herd in Alberta, Canada." Epidemiol Infect 129(1): 163-172.
5. Khaitsa ML, Bauer ML, Gibbs PS, Lardy GP, Doetkott D, Kegode RB (2005) "Comparison of two sampling methods for *Escherichia coli* O157:H7 detection in feedlot cattle." J Food Prot 68(8): 1724-1728.
6. Khaitsa ML, Bauer ML, Lardy GP, Doetkott DK, Kegode RB, Gibbs PS (2006) "Fecal shedding of *Escherichia coli* O157:H7 in North Dakota feedlot cattle in the fall and spring." J Food Prot 69(5): 1154-1158.
7. Khaitsa ML, Smith DR, Stoner JA, Parkhurst AM, Hinkley S, Klopfenstein TJ, Moxley RA (2003)"Incidence, duration, and prevalence of *Escherichia coli* O157:H7 fecal shedding by feedlot cattle during the finishing period." J Food Prot 66(11): 1972-1977.
8. Lahti E, Ruoho O, Rantala L, Hanninen M, Honkanen-Buzalski T (2003) "Longitudinal study of *Escherichia coli* O157 in a cattle finishing unit." Appl Environ Microbiol 69(1): 554-561.
9. Riley DG, Loneragan GH, Phillips WA, Gray JT, Fedorka-cray PJ (2008) "Fecal shedding of foodborne pathogens by Florida-born heifers and steers in U.S. beef production segments." J Food Prot 71(4): 807-810.
10. Sanderson MW, Sargeant JM, Shi X, Nagaraja TG, Zurek L, Alam MJ (2006) "Longitudinal emergence and distribution of *Escherichia coli* O157 genotypes in a beef feedlot." Appl Environ Microbiol 72(12): 7614-7619.
11. Thran BH, Hussein HS, Hall MR, Khaiboullina SF (2001) "Occurrence of verotoxin-producing *Escherichia coli* in dairy heifers grazing an irrigated pasture." Toxicology 159(3): 159-169.
12. Thran BH, Hussein HS, Hall MR, Khaiboullina SF (2001) "Shiga toxin-producing *Escherichia coli* in beef heifers grazing an irrigated pasture." J Food Prot 64(10): 1613-1616.
13. Tokhi AM, Peirisl JSM, Scotland SM, Willshaw GA, Saiith HR, Cheasty T (1993) "A longitudinal study of Vero cytotoxin producing *Escherichia coli* in cattle calves in Sri Lanka." Epidemiol Infect 110(2): 197-208.

**Same dataset in different publications (n=11)**

1. Aspan A, Eriksson E (2010) "Verotoxigenic *Escherichia coli* O157:H7 from Swedish cattle; isolates from prevalence studies versus strains linked to human infections--a retrospective study." BMC Vet Res 6: 7.
2. Blanco J, Blanco M, Blanco JE, Mora A, González EA, Bernárdez MI, Alonso MP, Coira A, Rodríguez A, Rey J, Alonso JM, Usera MA (2003) "Verotoxin-producing *Escherichia coli* in Spain: Prevalence, serotypes, and virulence genes of O157 : H7 and non-O157 VTEC in ruminants, raw beef products, and humans." Experimental Biology and Medicine 228(4): 345-351.
3. Dodd CC, Renter DG, Fox JT, Shi X, Sanderson MW, Nagaraja TG (2010) "Genetic relatedness of *Escherichia coli* O157 isolates from cattle feces and preintervention beef carcasses." Foodborne Pathog Dis 7(4): 357-365.
4. Fernandez D, Rodrıguez EM, Arroyo GH, Padola NL, Parma AE (2009) "Seasonal variation of Shiga toxin-encoding genes (stx) and detection of E. coli O157 in dairy cattle from Argentina." J Appl Microbiol 106(4): 1260-1267.
5. HERRIOTT DE, HANCOCK DD, EBEL ED, CARPENTER LV, RICE DH, BESSER TE (1998) “Association of Herd Management Factors with Colonization of Dairy Cattle by Shiga Toxin-Positive *Escherichia coli* 0157” Journal of Food Protection, 61 (7), 802-807.
6. Manna SK, Manna C, Batabyal K, Das B, Golder D, Chattopadhyay S, Biswas BK (2010) "Serogroup distribution and virulence characteristics of sorbitol-negative *Escherichia coli* from food and cattle stool." J Appl Microbiol 108(2): 658-665.
7. Matthews L, Mckendrick IJ, Ternent H, Gunn GJ, Synge B, Woolhouse MEJ (2006) "Super-shedding cattle and the transmission dynamics of *Escherichia coli* O157." Epidemiol Infect 134(1): 131-142.
8. Renter DG, Sargeant JM, Oberst RD, Samadpour M (2003) "Diversity, frequency, and persistence of *Escherichia coli* O157 strains from range cattle environments." Appl Environ Microbiol 69(1): 542-547.
9. Sargeant JM, Sandersonb MW, Smithc RA, Griffin DD (2004) "Associations between management, climate, and *Escherichia coli* O157 in the faeces of feedlot cattle in the Midwestern USA." Prev Vet Med 66(1-4): 175-206.
10. Sargeant JM, Shi X, Sanderson MW, Renter DG, Nagaraja TG (2006) "Pulsed-field gel electrophoresis patterns of *Escherichia coli* O157 isolates from Kansas feedlots." Foodborne Pathog Dis 3(3): 251-258.
11. Williams MS, Withee JL, Ebel ED, Bauer NE Jr., Schlosser WD, Disney WT, Smith DR, Moxley RA (2010) "Determining relationships between the seasonal occurrence of *Escherichia coli* O157:H7 in live cattle, ground beef, and humans." Foodborne Pathog Dis 7(10): 1247-1254.

**Sample source unknown (n=2)**

1. Hornitzky MA, Bettelheim KA, Djordjevic SP (2001) "The detection of Shiga toxin-producing *Escherichia coli* in diagnostic bovine faecal samples using vancomycin-cefixime-cefsulodin blood agar and PCR." FEMS Microbiol Lett 198(1): 17-22.
2. Hornitzky MA, Mercieca K, Bettelheim KA, Djordjevic SP (2005) "Bovine feces from animals with gastrointestinal infections are a source of serologically diverse atypical enteropathogenic *Escherichia coli* and Shiga toxin-producing E. coli strains that commonly possess intimin." Appl Environ Microbiol 71(7): 3405-3412.

**Others (n=10)**

1. Gansheroff LJ, O'Brien AD (2000) "*Escherichia coli* O157:H7 in beef cattle presented for slaughter in the U.S.: higher prevalence rates than previously estimated." Proc Natl Acad Sci U S A 97(7): 2959-2961.
2. Gioffré A, Meichtrib L, Miliwebskyc L, Baschkierc A, Chillemic G, Romanoa MI, Estanid SS, Cataldia A, Rodrıguezb R,Rivas M (2002) "Detection of Shiga toxin-producing *Escherichia coli* by PCR in cattle in Argentina: Evaluation of two procedures." Vet Microbiol 87(4): 301-313.
3. Hu Y, Zhang Q, Meitzler JC (1999) "Rapid and sensitive detection of *Escherichia coli* O157:H7 in bovine faeces by a multiplex PCR." J Appl Microbiol 87(6): 867-876.
4. Kagambèga A, Martikainen O, Siitonen A, Traore AS, Barro N, Haukka K (2012) "Prevalence of diarrheagenic *Escherichia coli* virulence genes in the feces of slaughtered cattle, chickens, and pigs in Burkina Faso." MicrobiologyOpen 1(3): 276-284.
5. Rogerie F, Marecata A, Gambadeb SP, Duponda F, Beauboisb P, Langea M (2001) "Characterization of Shiga toxin producing E. coli and O157 serotype E. coli isolated in France from healthy domestic cattle." Int J Food Microbiol 63(3): 217-223.
6. Sharma R, Stanford K, Louie M, Munns K, John SJ, Zhang Y, Gannon V, Chui L, Read R, Topp E, Mcallister T (2009) "*Escherichia coli* O157:H7 lineages in healthy beef and dairy cattle and clinical human cases in Alberta, Canada." J Food Prot 72(3): 601-607.
7. Shaw DJ, Jenkins C, Pearce MC, Cheasty T, Gunn GJ, Dougan G, Smith HR, Woolhouse MEJ, Frankel G (2004) "Shedding patterns of verocytotoxin-producing *Escherichia coli* strains in a cohort of calves and their dams on a Scottish beef farm." Appl Environ Microbiol 70(12): 7456-7465.
8. Synge BA, Chase-topping ME, Hopkins GF, Mckendrick IJ, Thomson-carter F, Gray D, Rusbridge SM, Munro FM, Foster G, Gunn GJ (2003) "Factors influencing the shedding of verocytotoxin-producing *Escherichia coli* O157 by beef suckler cows." Epidemiol Infect 130(2): 301-312.
9. Vidovic S, Germidab JJ, Korbera DR (2007) "Sensitivity of two techniques to detect *Escherichia coli* O157 in naturally infected bovine fecal samples." Food Microbiol 24(6): 633-639.
10. Wilson JB, McEwen SA, Clarke RC, Leslie KE, Wvilson RA, Waltner-toewas D, Gyles CL (1992) "Distribution and characteristics of verocytotoxigenic *Escherichia coli* isolated from Ontario dairy cattle." Epidemiol Infect 108(3): 423-439.
